# Fluorescent Sensor Based on 1*H*-Pyrazolo[3,4-*b*]quinoline Derivative for Detecting Zn^2+^ Cations

**DOI:** 10.3390/molecules29040823

**Published:** 2024-02-10

**Authors:** Anna Kolbus, Tomasz Uchacz, Andrzej Danel, Katarzyna Gałczyńska, Paulina Moskwa, Przemysław Kolek

**Affiliations:** 1 Institute of Chemistry, The Jan Kochanowski University, Uniwersytecka 7 St., 25-406 Kielce, Poland; anna.kolbus@ujk.edu.pl (A.K.); paulinam1999@tlen.pl (P.M.); 2 Faculty of Chemistry, Jagiellonian University, Gronostajowa 2 St., 30-387 Kraków, Poland; 3 Faculty of Materials Engineering and Physics, Cracow University of Technology, Podchorążych St.1, 30-348 Kraków, Poland; rrdanela@cyf-kr.edu.pl; 4 Institute of Biology, The Jan Kochanowski University, Uniwersytecka 7 St., 25-406 Kielce, Poland; kgalczynska@ujk.edu.pl; 5 Institute of Physics, University of Rzeszów, 1 Pigonia St., 35-310 Rzeszów, Poland; pkolek@ur.edu.pl

**Keywords:** fluorescent sensor, pyrazoloquinoline derivative, DFT calculation, Zn^2+^ detection

## Abstract

The photophysical and sensory properties of the donor–acceptor pyrazoloquinoline derivative (PQPc) were investigated using absorption, steady-state, and time-resolved fluorescence measurements. The compound synthesized from commercial, readily available substrates exhibited absorptions in the UV–Vis range, with a maximum of the longwave band around 390 nm. The maximum fluorescence was around 460–480 nm, depending on the solvent. The quantum yield was between 12.87% (for *n*-hexane) and 0.75% (for acetonitrile) and decreased with increasing solvent polarity. The PET mechanism was implicated as the cause of fluorescence quenching. Divalent ions such as Zn^2+^, Pb^2+^, Cd^2+^, Ca^2+^, Mg^2+^, Co^2+^, Ni^2+^, and Cu^2+^ were introduced to study the fluorescent response of PQPc. A 13-times increase in fluorescence quantum yield was observed after the addition of Zn^2+^ ions. Detailed research was carried out for the PQPc-Zn^2+^ system in order to check the possibility of analytical applications of PQPc as a fluorescent sensor. A detection limit of Zn^2+^ was set at the value level 1.93 × 10^−7^ M. PQPc-Zn^2+^ complexes had a stoichiometry of 1:1 with a binding constant of 859 M^−1^. Biological studies showed that the sensor was localized in cells near the membrane and cytoplasm and may be used to detect zinc ions in eukaryotic cells.

## 1. Introduction

**Figure 1:**
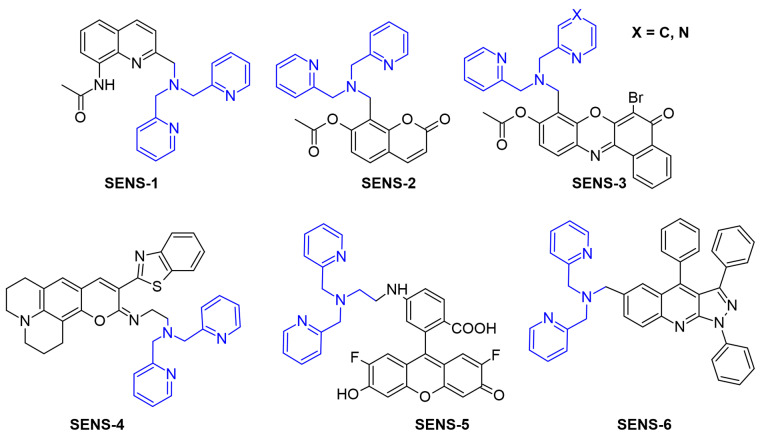
Some fluorescent sensors with dipicolylamine as the recognition center.

The world of living nature surrounding us, including plant, animal, and human organisms, functions owing to the entire cycle of biochemical transformations, which in many cases depend on the presence of a whole range of macro- and microelements [1,2,3]. There is a whole range of cations that belong to the main group elements of the periodic table as well as to the transition elements. The first group includes macroelements, which include, among others, potassium, which affects the body’s water management and circulatory system. Another is magnesium, which is a key building block of bones and teeth and is responsible for reducing the risk of heart disease, reducing stress, and relieving insomnia. It is also worth mentioning calcium, which is found practically quantitatively in our bones and teeth, as well as in soft tissues and body fluids. The second group is microelements, including transition metals such as iron, copper, and zinc. Iron is responsible for the processes of breathing and the formation of red blood cells. Copper, in turn, is involved in the synthesis of hemoglobin and cholesterol metabolism. The third of the mentioned elements, zinc, plays a catalytic, structural, and regulatory role [4]. In the second half of the 19th century, it was discovered that zinc is necessary for the proper development of plants. In 1934 and 1961 it was shown that this element is also necessary for the proper development of animals and humans [5]. Zinc is involved in the action of over 300 enzymes that catalyze hydrolysis reactions or the transport of functional groups [6,7]. The daily requirement for zinc, depending on age, ranges from 2 mg for infants to 11–13 mg for adults, including pregnant and breastfeeding women [8]. Lack of zinc in the body results in, among others, loss of appetite, nervousness, and anxiety. In men, spermatogenesis and the synthesis of steroid hormones may be impaired. In children, a slowdown in growth and maturation is observed [9,10]. Of course, it should be remembered that an excess of zinc and other microelements may be harmful, according to the Paracelsus principle—everything is a poison and nothing is a poison, it is the dose that makes the poison. For example, employees working in zinc smelters are exposed to excessive doses of zinc, where they suffer from an occupational disease called foundry fever [6,11,12]. Based on these few examples, it can be seen that controlling the level of zinc as well as a whole range of macro- and microelements is necessary for the proper functioning of living organisms. In the case of people and animals, it may be necessary to supplement these elements, and fertilization is necessary for the proper development of plants. Therefore, the question arises of how to test the content and presence of zinc ions and other previously mentioned cations. There is a whole range of analytical techniques, some of the most important of which include those based on the phenomenon of fluorescence. Fluorescent molecular sensors for ions (e.g., Na^+^, K^+^, Zn^2+^, and Ca^2+^), neutral molecules (e.g., simple sugars, explosives, drugs, and amino acids), or gases (NO and oxygen) have an established position in analytical chemistry [13]. The above-mentioned sensors most often work based on the mechanism of photoinduced charge transfer PCT or photoinduced electron transfer PET. This last category includes, among others, turn-on sensors, which consist of a heterocyclic fluorophore connected via a spacer to a dipicolylamine system as a recognition group. Excitation of the fluorophore causes an electron to transfer from the highest occupied molecular orbital (HOMO) level of the donor dipicolylamine to the lowest unoccupied molecular orbital (LUMO) level of the acceptor 1,3-diphenyl-pyrazoloquinoline. This mechanism causes fluorescence quenching. In the case of a positive reaction to the cation, an enhancement of the emission is observed. Then, the HOMO energy decreases so much in relation to the fluorophore energy that PET does not occur and fluorescence quenching is suppressed [13,14,15]. These sensors are applied for the detection of zinc ions. Wang and colleagues described the SENS-1 zinc sensor based on 8-aminoquinoline and dipicolylamine at position 2 of the parent system. The obtained system turned out to be sensitive to zinc within the range of 2.15 × 10^−9^ M. Additionally, the presence of cadmium ions did not interfere with the detection of zinc ions [16]. Lippard’s group synthesized two sensors based on dipicolylamine modified with acetyl groups for multicolor imaging, which emitted blue light SENS-2 or red light SENS-3 in the presence of zinc. It was shown that the acetylation of hydroxyl groups in the parent systems increased the level of sensor sensitivity [17]. Another interesting example of a sensor with dipicolylamine as a recognition group is the 2-iminocoumarin derivative SENS-4. The compound is characterized by high emission quantum yield in an aqueous environment after excitation with visible light. After the complexation of Zn^2+^ cations, a redshift of the emission is observed [18]. Research on a fluorescein derivative as a potential fluorescent zinc sensor was a great success. Free compounds have a very low quantum yield of 0.004–0.006 (depending on the position of dipicolylamine in the phenyl ring). After zinc complexation, the emission is enhanced 60–69 times. Both compounds are commercially available [19]. 1*H*-pyrazolo[3,4-*b*]quinolines exhibit high quantum yields of fluorescence so they were applied among others as luminophores for Organic Light Emitting Diodes and as fluorescent probes as well [20,21]. Last but not least is the sensor based on 1*H*-pyrazolo[3,4-*b*]quinoline SENS-6 [22]. The sensor SENS-6 appeared to be sensitive toward Zn^2+^ and Mg^2+^ cations. It forms complexes both in ratio SENS-6:M^n+^ and (SENS-6)_2_:M^n+^. Apart from the previously described sensing mechanisms, in the literature one can find many others that take advantage of physical phenomena like fluorescence resonance energy transfer [23], excited-state proton transfer [24,25], aggregation-induced emission [26], and excimer/exciplex formation [27,28]. The mentioned earlier fluorescent probes are depicted in Figure 1. Fluorescent sensors based on the PET mechanism can be used to design logic gates [14,29]. Easily detectable fluorescence and a wide spectrum of used ions allow the creation of various types of logic gates using Boolean logic, such as AND [30,31,32], OR [33,34], and INHIBIT [30,34,35,36] logic gates.

The goal of this paper is to investigate the sensing properties of 1,3-diphenyl-1*H*-pyrazolo[3,4-*b*]quinoline with dipicolylamine recognition at the fourth position in the pyridine ring of the parent skeleton (PQPc). The dipicolylamine group is commonly known as a good chelator for Zn^2+^. Hence, in our experiments, we mainly concentrated on the detection of bivalent cations. We also tested the performance of our sensor in biological systems, i.e., in eukaryotic cells.

## 2. Results and Discussion

### 2.1. Spectroscopic Studies

**Figure 2:**
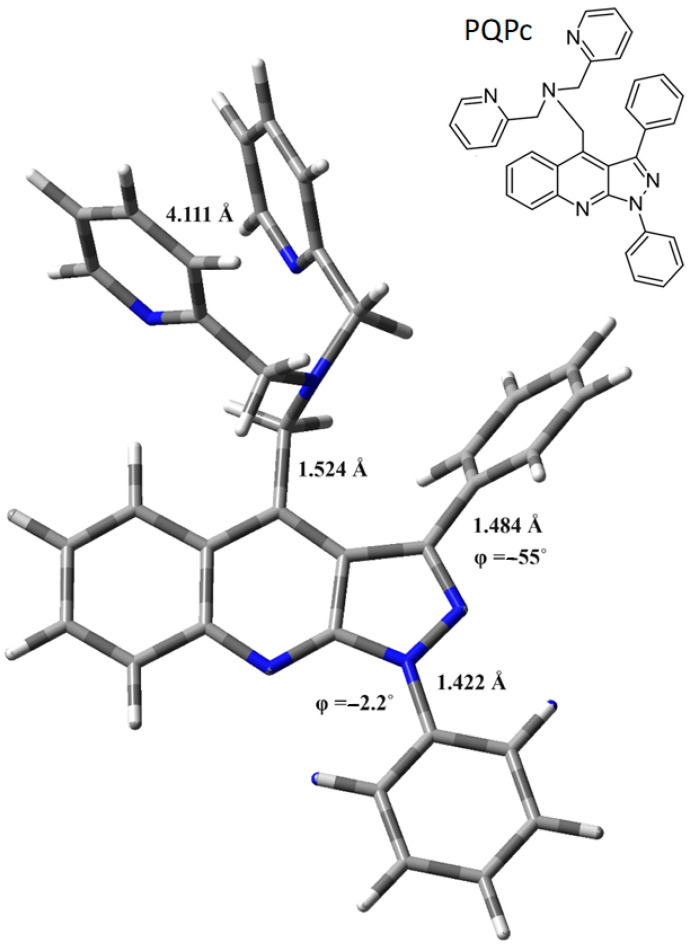
Optimized molecular geometry of PQPc calculated by the DFT/B3LYP cc-pVDZ method along with selected bond lengths and interplanar angles.

The molecular structure of the studied sensor was optimized by DFT calculation and presented in Figure 2.

The main skeleton of PQPc adopts the conformation with phenyl at the first position being in the plane of the core (φ = −2.2°) and twisted out of the plane phenyl at the third position (φ = −55°). The receptor part with almost parallel pyridines in the ground state is situated above the plane of the pyrazoloquinoline. A relatively short distance between the pyridines’ planes of dipicolylamine (4.111 Å) may favor the existence of sandwich-like π-π interactions. The effect of polarity (acetonitrile) on the relaxation of conformation can be neglected since the three-phenyl ring is twisted by a torsion angle of φ = −57°, and pyridine planes increased the distance up to 4.144 Å in acetonitrile. Studying the bond lengths between the substituents and the pyrazoloquinoline core it is crucial to note that the shortest length is observed for N–C at the first position 1.422 Å, then for C–C at the third position 1.484 Å, and finally 1.524 Å for C–C at the fourth position. The first two values do not deviate much from those reported for 6-ethyl-1,3-diphenyl-pyrazoloquinoline [37], showing electronic coupling occurring between phenyl rings and the core. In the case of the substituent at the fourth position, a clear single molecular bond is observed pointing to a lack of electronic conjugation between the receptor and the chromophore.

**Figure 3:**
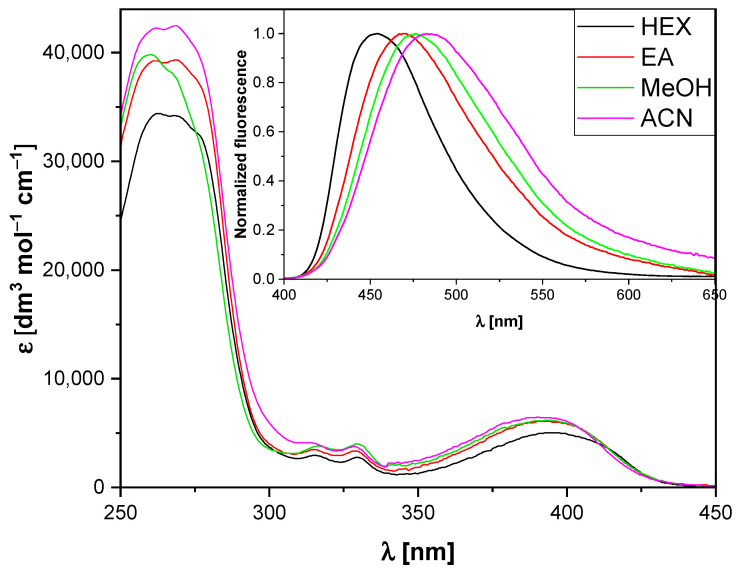
Absorption and corrected and normalized fluorescence (inset) spectra of PQPc in a few solvents of different polarity. The concentration of PQPc in each solvent was 1.73 × 10^–5^ M. The sample was excited at 380 nm.

The photophysical properties of the studied compound were investigated in several organic solvents of different polarity: *n*-hexane (HEX), diethyl ether (DEE), ethyl acetate (EA), tetrahydrofuran (THF), methanol (MeOH), and acetonitrile (ACN). The absorption spectra of PQPc in these solvents are shown in Figure 3. The positions of absorption and fluorescence intensity maxima, along with several basic photophysical parameters, are collected in Table 1.

The absorption spectrum consists of two bands: a structured band between 250 nm and 300 nm and a Gaussian-like broad band between 350 nm and 450 nm with a maximum of about 390 nm. The absorption maximum is weakly blue-shifted with increasing solvent polarity, which is a typical feature of the parent molecule (1,3-diphenyl-pyrazoloquinoline) and accounted for the coexistence of at least two rotamers with phenyl-twisted substituents [37] (see Table 1).

The fluorescence spectra are presented in Figure 3 (inset). The sample was excited at 380 nm. The bathochromic shift of the fluorescence maximum with increasing solvent polarity is observed, which may point to the presence of an excited state with a charge transfer character. Such a state represented by a molecule with a higher dipole moment (compared with the ground state) is stabilized in highly polar solvents. Hence, the noticed redshift of fluorescence maximum can be justified. Moreover, this behavior is peculiar for the phenyl-substituted pyrazoloquinolines, showing a predominant effect of phenyl substituent at the first position on the charge transfer character of the first singlet excited state [38].

**Figure 4:**
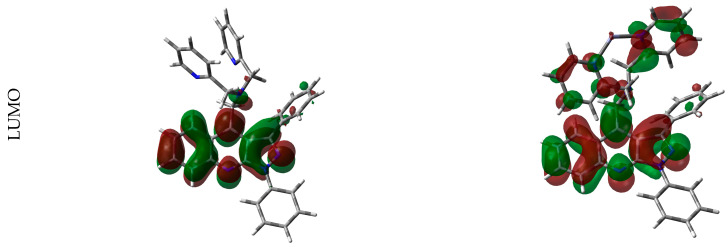

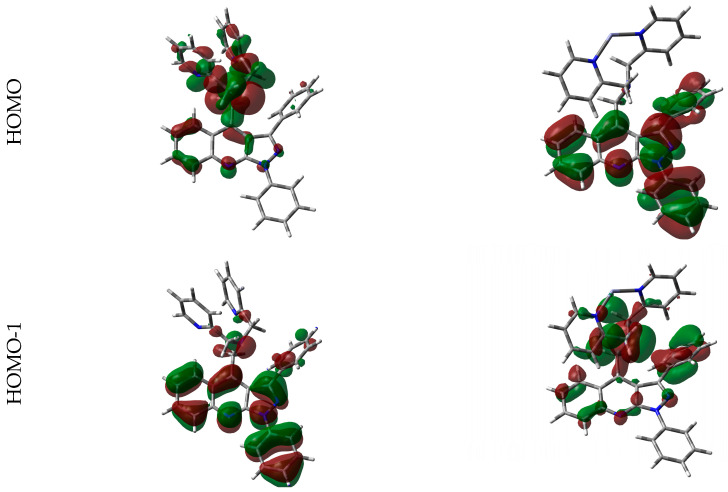
HOMO-1, HOMO, and LUMO orbitals of PQPc and for the complex PQPc with Zn^2+^.

The existence of a charge transfer process occurring within the pyrazoloquinoline core was corroborated by time-dependent density functional theory calculations (TD-DFT, B3LYP/cc-pVDZ, and level of theory). The HOMO and LUMO orbitals and DFT-predicted absorption spectra in the ACN solvent were reproduced using the program Gaussview and are displayed for both dyes in Figure 4 and Figure S1, respectively. Our DFT calculations revealed that the first absorption band of the sensor consists of two transitions: the HOMO–LUMO (λ_abs_ = 449 nm) and the HOMO-1 → LUMO (λ_abs_ = 407 nm). Since the oscillator strength of the HOMO–LUMO transition is very low (*f* = 0.0042), it can be concluded that the HOMO-1 → LUMO transition (*f* = 0.1258) is mainly responsible for light absorption in the UV–Vis region. It should be stressed here that the HOMO-1 → LUMO transition occurs only within the 1,3-diphenyl-pyrazoloquinoline subunit and is related to a charge relocation from phenyl substituents to the pyrazoloquinoline core. In the case of the HOMO–LUMO transition, the charge is transferred from the receptor part to the chromophore core. Moreover, the contours of HOMO orbitals, in particular the large electron density located on the parallelly oriented pyridines, may partially confirm the above-postulated hypothesis on the existence of sandwich-like π-π interactions. Finally, it is worth mentioning that the energy of the vertical transition is slightly underestimated (0.13 eV), but the shape of the absorption spectrum is well reproduced (see Figure S1). On the other hand, however, the energy of the DFT-predicted emission transition is slightly overestimated (see Figure S2). It is worth adding that the charge distribution of the HOMO orbitals for the molecule in the excited state geometry differs from the molecule in the ground state geometry (Figure S3). Here, the charge density is fully located at the receptor part, indicating that the receptor may play a crucial role in interactions with metal cations and can be alternatively identified as the HOMO orbital of the electron donor (dipicolylamine) itself. The observed energy arrangement of the orbitals is favorable in terms of the efficiency of the photoinduced electron transfer process. From the theory, it follows that electron transfer is possible if the electron donor appears in close proximity to the excited chromophore and its HOMO energy is lower than that of the donor’s HOMO level. Such energy arrangement enables the electron injection to the valence band of the excited PQPc leading to the formation of a cation–anion radical pair within the molecule. The predicted DFT energy of the HOMO–LUMO transition is very low, 704 nm, pointing out that these radicals may rapidly recombine with each other in highly polar solvents (acetonitrile), leading to the recovery of the ground state of the dye. This analysis fully correlates with the observed low fluorescence quantum yield of PQPc in acetonitrile.

The highest value of fluorescence quantum yield occurs for PQPc in non-polar *n-*hexane while the lowest is in acetonitrile. This behavior suggests the existence of the process strongly polarity dependent, which can efficiently quench the fluorescence in polar solvents. Based on our previous research related to the pyrazoloquinoline-based sensor with dipicolylamine at the sixth position attached via the methylene group [22], one can speculate on the existence of a similar deactivation mechanism of the first excited state, i.e., photoinduced electron transfer process. This process occurring between the electron donor, dipicolylamine, and the electron acceptor, 1,3-diphenyl-pyrazoloquinoline, can be easily inhibited by the chelation of the analyte, which leads to the recovery of the native fluorescence of the chromophore. Such behavior lays the foundation of OFF–ON fluorescence sensors [39].

### 2.2. Bivalent Cation Sensing Studies of PQPc

**Figure 5:**
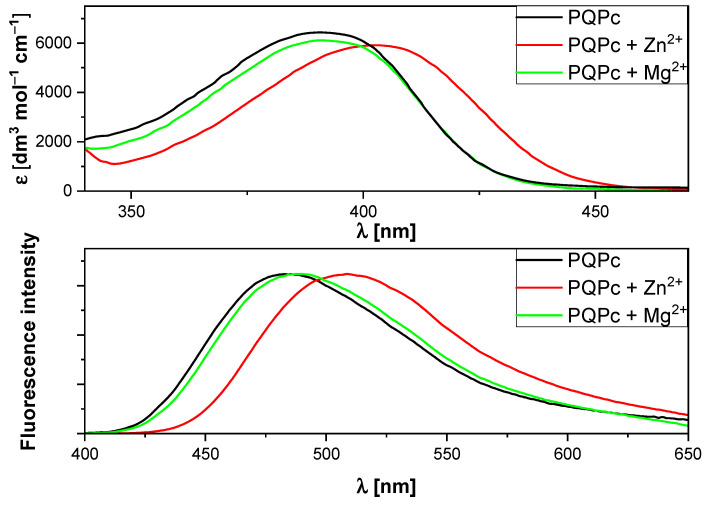
Absorbance (**top**) and fluorescence (**bottom**) spectra of PQPc in acetonitrile without ions, and with Zn^2+^ and Mg^2+^ ions. The fluorescence spectra were normalized to the first absorption band of PQPc in acetonitrile. The concentration of PQPc was 1.73 × 10^–5^ M, and the concentrations of Zn^2+^ and Mg^2+^ were 1.37 × 10^–5^ M. The sample was excited at 380 nm.

The fluorescence response of PQPc to the selected ions like Zn^2+^, Cd^2+^, Mg^2+^, Co^2+^, Ni^2+^, Ca^2+^, Pb^2+^, and Cu^2+^ was checked in acetonitrile solution. Studies of bivalent cations result from previous experience with the sensory properties of pyrazoloquinoline derivatives [40]. The experiment was performed in acetonitrile due to the most efficient PET process observed in this solvent and the high solubility of the cations in polar solvents. The initial concentration of the dye was 1.73 × 10^−5^ M. When a solution of ion was added, both the absorption maximum (Δλ_abs_ = 13 nm in the presence of Zn^2+^) and fluorescence maximum shifted bathochromically (Figure 5). The maximum fluorescence shift of approximately 20–30 nm occurs after the addition of Zn^2+^, Pb^2+^, and Cd^2+^, smaller shifts—approximately 10 nm—are noticeable for Ca^2+^ and Mg^2+^, while no maximum fluorescence shift is visible for Co^2+^, Cu^2+^, and Ni^2+^.

**Figure 6:**
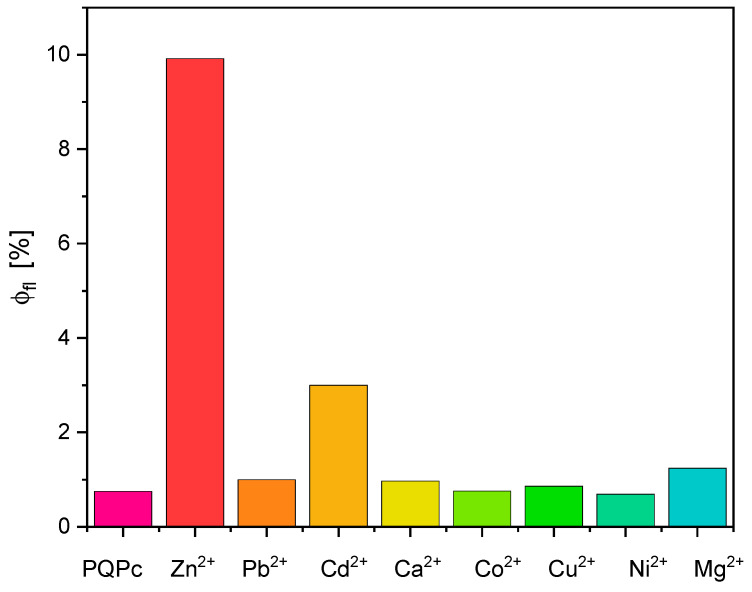
The fluorescence quantum yield values for PQPc in the presence of different metal ions. The ion concentrations in the graph are 1.37 × 10^−5^ M.

Figure 6 shows the fluorescence quantum yield values for the PQPc sample in acetonitrile after adding a solution of the studied ions at a concentration of 1.37 × 10^−5^ M. The highest fluorescence quantum yield was obtained for the PQPc-Zn^2+^ complex. The quantum yield increased from 0.75% for the PQPc sample without the addition of ions to 9.92% after the addition of Zn^2+^ ions, which means an approximately 13-fold increase in the quantum yield for the PQPc-Zn^2+^ system compared with the PQPc. Lower values of 3.00% and 1.25% were obtained for PQPc complexes with Cd^2+^ and Mg^2+^, respectively. The addition of the remaining ions gave a quantum yield below 1%. Therefore, in further analysis, we focused on studying the PQPc-Zn^2+^ system.

### 2.3. Sensory Properties of PQPc toward Zn^2+^ Ions

**Figure 7:**
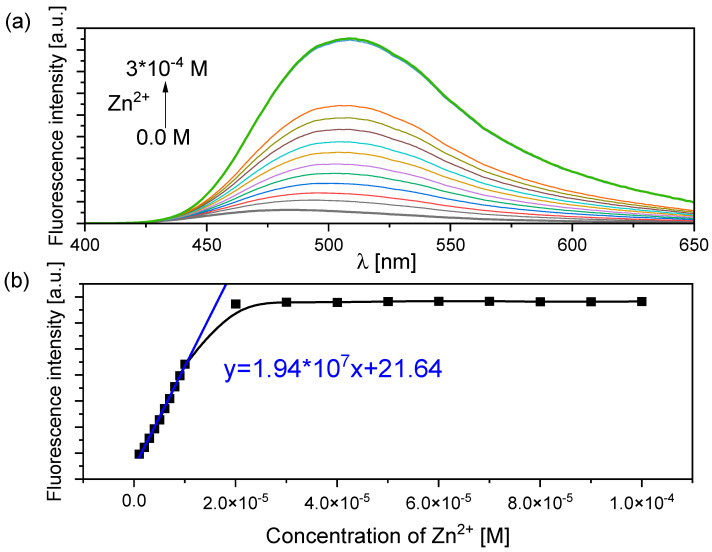
Fluorescence spectra of PQPc in acetonitrile in the absence and presence of zinc nitrate (**a**) and the dependence of the fluorescence intensity on the concentration of cations for Zn^2+^ at 490 nm (**b**). The concentration of the Zn^2+^ spans the region of 0.0–3 × 10^–5^ M.

Fluorescence titration of the PQPc sensor solution was carried out using a solution of Zn^2+^ ions in the concentration range of 1 × 10^− 6^ to 1 × 10^−4^. Fluorescence spectra of PQPc in acetonitrile in the absence and presence of zinc nitrate are shown in Figure 7a. In the absence of ions, PQPc shows a weak emission band centered around 483 nm. After adding the Zn^2+^ solution, the band maximum shifts to 510 nm. The maximum value of fluorescence quantum yield was achieved at a concentration of Zn^2+^ ions equal to 2 × 10^−5^ M (ϕ_fl_ = 10%) and further increasing the ion concentration did not result in a significant increase in quantum yield and did not shift the fluorescence maximum. A linear relationship between the concentration of zinc ions and the fluorescence intensity is visible at a ratio of $c_{Zn^{2+}}/c_{PQPc}$ < 1.2 (Figure 7b). The linear range between the concentration of Zn^2+^ ions and fluorescence intensity in the titration curve was used to determine the limit of detection for Zn^2+^ ions. Using the formula $\mathrm{LOD}=\frac{3\cdot \mathrm{standard}\;\mathrm{deviation}\;\mathrm{of}\;\mathrm{blank}\;\mathrm{sample}}{\mathrm{slope}}$, the LOD of Zn^2+^ was estimated to be 1.93 × 10^−7^ M and is approximately 90 times lower than the ligand concentration. This is a lower LOD value than for the pyrazoloquinoline derivative with crown moiety, which was 5.97 × 10^−6^ M [41], and similar to the LOD of the derivative with a diethylamine substituent, which was equal to 4 × 10^−7^ M [40].

### 2.4. Binding Stoichiometry and Sensing Mechanism

**Figure 8:**
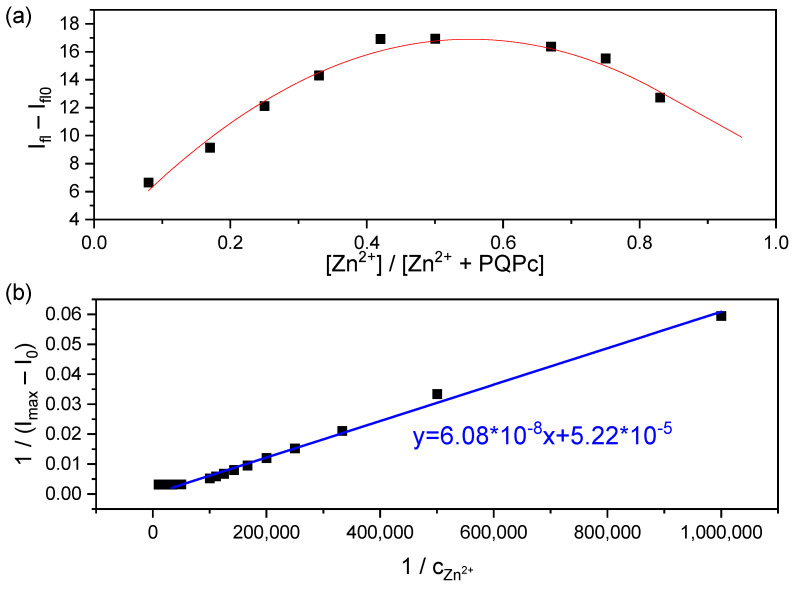
The Job’s plot of Zn^2+^ versus ([PQPc] + [Zn^2+^] = 17 μM) at 490 nm emission wavelength (**a**) and determination of the binding constant of PQPc complexes with Zn^2+^ ions (**b**). The solid lines present the fitted functions to the experimental data.

The Job’s method was applied to estimate the stoichiometry of the PQPc-Zn^2+^ complex. It is seen that the complex of 1:1 stoichiometry is formed in the ground state. The maximum on the Job curve is flattened (Figure 8a), so a small constant stability of the complex is expected. For the complex with 1:1 stoichiometry, the Benesi–Hildebrand method was used to determine the binding constant [42]. The binding constant for the PQPc complex with Zn^2+^ cation was estimated to be 859 M^−1^ (Figure 8b). This value is comparable to the complexes formed by the derivative with a crown moiety (978 M^−1^) [41], but much lower compared with the complexes formed with divalent ions by the derivative with a diethylamine substituent (the binding constant of the order of magnitude 10^3^–10^10^ M^−1^) [40].

**Scheme 1:**
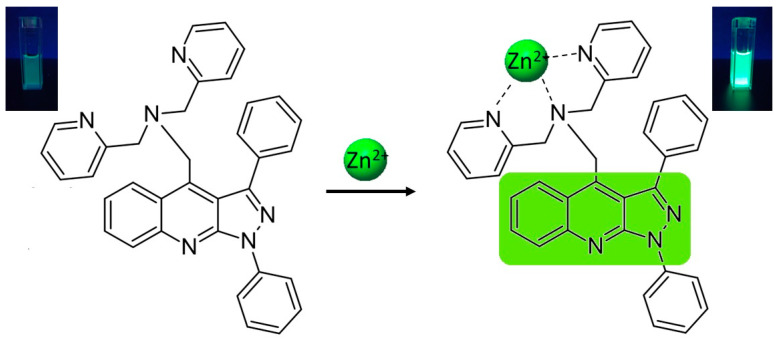
The proposed working mechanism of the PQPc sensor toward the Zn^2+^ cation. Green box was used to show the color of emitted light.

The proposed working mechanism of the PQPc sensor toward the Zn^2+^ cation is shown in Scheme 1. It was assumed that the electron transfer process in the PQPc molecule causes fluorescence quenching. The specific electrostatic interactions between the cation and the dipicolylamine substituent lead to the suppression of the electron transfer process and, consequently, to a recovery of fluorescence from the local excited state.

To provide better insight into the sensor–Zn^2+^ interactions and their photophysical properties, the DFT and TDDFT calculations were performed. The contours of molecular orbitals are presented in Figure 4, and the simulated absorption spectra are displayed in Figure S1. In the case of the complex PQPc-Zn^2+^, the first absorption band is dominated by the HOMO–LUMO transition, giving a 96% contribution to the electronic S_0_ → S_1_ transition (λ_abs_ = 436 nm, *f* = 0.110). Evidently, the S_0_ → S_1_ transition is accompanied by charge relocation from the phenyl substituents mainly to the core and partly to the receptor, the dipicolylamine moiety (see Figure 4). The energy of the HOMO-1 → LUMO transition was found to be 3.53 eV (λ_abs_ = 351 nm, *f* = 0.005, fifth excited state) and cannot compete with the HOMO–LUMO transition as it was observed for the free ligand. Hence, it can be concluded that the absorption and emission of the complex with Zn^2+^ originate from the locally excited state of the ligand PQPc because the HOMO → LUMO transition of the complex has a similar character (charge relocation) to the HOMO-1 → LUMO transition of the ligand. The simulated UV–Vis spectra for the complex agree well with the experimental data (Figure S1), correctly reproducing the experimentally observed redshift of the absorption maximum and decreasing the molar absorption coefficient in the lowest energy spectral region. Although the energy of the vertical transition is again slightly underestimated, the redshift trend seems to be well reflected. The energy of the DFT-predicted emission transition is slightly underestimated compared with the experiment; however, the deviation is not as large as in the absorption spectra (see Figure S2). Likewise in absorption, the emission band of the complex is dominated by the LUMO–HOMO transition with a 98% contribution to the S_1_ → S_0_ transition (λ_abs_ = 530 nm, *f* = 0.090). It should be stressed that the charge density of HOMO and LUMO orbitals calculated for the complex in excited state geometry is fully located at the chromophore core and does not appear at the receptor part (see Figure S4). This points out that the photoinduced electron transfer process is indeed inhibiting after Zn^2+^ binding.

**Figure 9:**
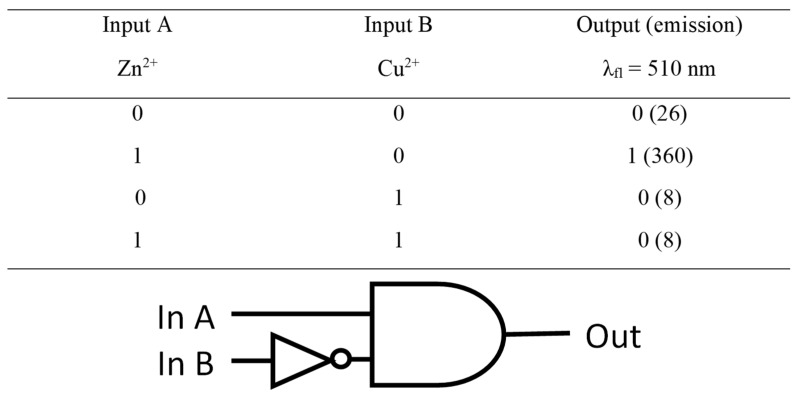
Truth table and schematic representation of INHIBIT logic gate. In parentheses, the relative fluorescence intensity for each channel is given. For each output channel, a threshold of 50% of the respective highest signal was applied. [Zn^2+^] = [Cu^2+^] = 1.35 × 10^−5^ M, [PQPC] = 1.73 × 10^−5^ M, λexc = 380 nm.

The competitive experiments were carried out by adding the Zn^2+^ solution to the PQPc solution in the presence of other cations. Both the concentration of the zinc ion and the remaining ions added were equal to 1.36 × 10^−4^ M. As can be observed in Figure S5, there is a high selectivity for Zn^2+^ detection in the presence of Pb^2+^, Ca^2+^, Cd^2+^, and Mg^2+^ ions. The introduction of a dipicolylamine substituent at the fourth position in the pyridine ring of the parent skeleton gave a pyrazoloquinoline derivative with much better selective properties than the previously studied derivative with a crown moiety at the fourth position. PQPc gives a clear reaction to one specific cation—Zn^2+^, while the derivative with a crown moiety showed weaker selectivity toward the studied ions [41]. However, in the presence of Co^2+^, Cu^2+^, and Ni^2+^ ions, the fluorescence of the Zn^2+^-PQPc complex is quenched. This may be the cause of the transfer of excitation energy from the ligand to the metal d-orbital [43]. Considering these results, a logic gate with Zn^2+^ and Cu^2+^ as inputs and the emission intensity at 510 nm as output, was tested. The signal was “1” only when the Zn^2+^ ion was present alone in the PQPc solution. There was an output “0” when Cu^2+^ was alone or both ions, Cu^2+^ and Zn^2+^, were present in the PQPc solution. The truth table and logic diagram for the INHIBIT function [44] are given in Figure 9.

The INHIBIT logic gate implements an integration of NOT and AND Boolean operations in one electronic molecular device. The NOT operation is only applied to one input, in this case to the presence of Cu^2+^ (Input B). Input B is a disabling input that turns off the output regardless of the presence of input A. In other words, INHIBIT logic has one input holding a veto over the other [15].

### 2.5. Biological Application Study

**Figure 10:**
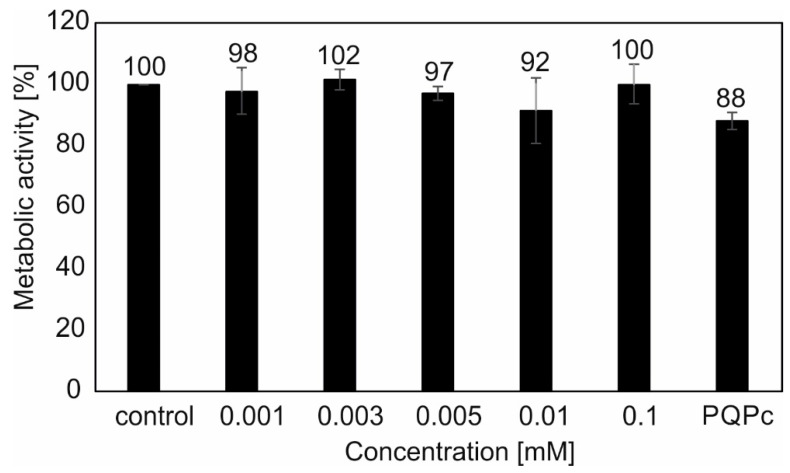
The metabolic activity of CHO-K1 cells treated with zinc(II) ions for 24 h was determined using the MTS test.

Cytotoxicity tests of zinc(II) ions using the MTS test showed that it does not affect the metabolic activity of CHO-K1 cells in the tested concentration range. The sensor slightly reduces the metabolic activity of cells after 24 h of incubation (Figure 10).

**Figure 11:**
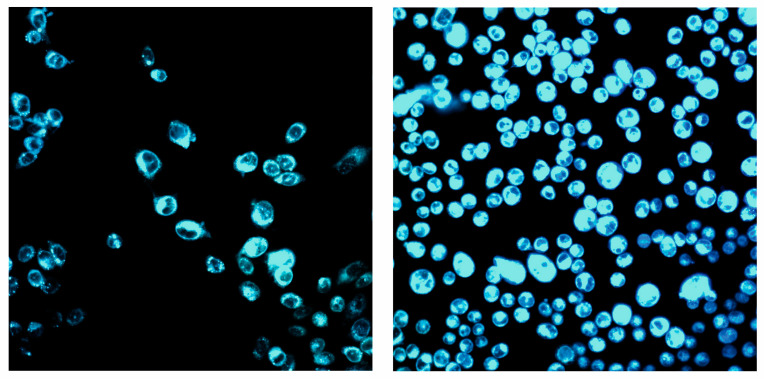
Confocal microscope photo of CHO-K1 cells treated with the PQPc sensor (**left**) and Zn^2+^ with the PQPc sensor (**right**) for 24 h.

After incubating the cells with zinc(II) ions, an attempt was made to detect zinc in the cells using the PQPc sensor. The experiment revealed only a slight increase in fluorescence intensity for the cells treated with zinc ions together with the sensor compared with the control (untreated cells) or cells with the sensor alone (Figure S6). Moreover, the confocal microscope studies showed that the sensor localizes in cells near the membrane and cytoplasm (Figure 11). Obviously, the observed fluorescence enhancement may be considered statistically insignificant, but the increase was clearly visible. Such behavior may be interpreted in terms of different locations of Zn^2+^ and PQPc storage in biological cells. It was scientifically proven that zinc cations are mostly stored in the nucleus, endoplasmic reticulum, Golgi apparatus, mitochondria, lysosomes, or zinc-binding proteins [45,46]. It seems that after 24 h of incubation, the concentration of Zn^2+^ in the cytoplasm or membrane may be relatively small due to the predominant effect of transportation and storage of Zn^2+^ in the above-mentioned cell organelles. Since our sensor was mostly located in the cytoplasm and membrane, the observed small fluorescence enhancement may be justified. Nevertheless, the behavior needs further exploration, especially in terms of the reduction in Zn^2+^ incubation time or the application of a higher Zn^2+^ concentration. Such experiments will be held in our laboratory in due course.

## 3. Materials and Methods

### 3.1. Synthesis

**Scheme 2:**
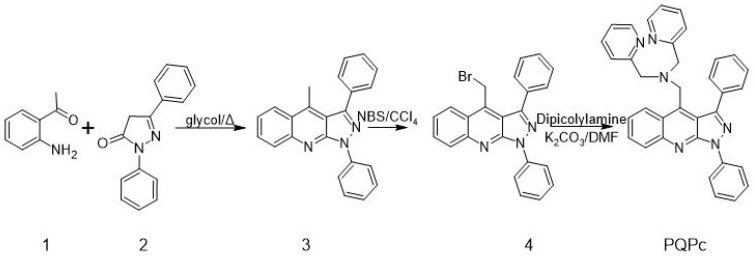
Synthesis of dipicolylamine substituted 1*H*-pyrazolo[3,4-*b*]quinoline PQPc.

The investigated sensor PQPc was synthesized according to Scheme 2. Thus, the starting 4-methyl-1,3-diphenyl-1*H*-pyrazolo[3,4-*b*]quinoline **3** was synthesized using a Friedländer condensation between *o*-aminoacetophenone **1** and 2,5-diphenyl-2,4-dihydro-3*H*-pyrazol-3-one **2**. Compound **3** was brominated with NBS in boiling carbon tetrachloride. The resulting bromoderivative 4 was subjected to the reaction with dipicolylamine in the presence of anhydrous potassium carbonate resulting in the final compound, PQPc. The syntheses of **3** and **4** were described in our previous paper [41].

#### The Synthesis of Derivative PQPc

A round-bottomed flask (50 mL) equipped with a magnetic stirring bar and reflux condenser was used for the experiment. The flask was filled with pyrazolo[3,4-*b*]quinoline **4** (300 mg, 0.7 mmol), dipicolylamine (150 mg, 0.75 mmol), anhydrous potassium carbonate (180 mg, 1.3 mmol), and 30 mL of acetonitrile. The content was refluxed for 2 h and filtered to remove inorganic residues. The filtrate was cooled and the resulting precipitate was filtered off and dried. Pale yellow crystals, 300 mg, 79.5%. ^1^H NMR (400 MHz, CDCl_3_, δ ppm): 8.54 (d, *J* = 7.8 Hz, 2H), 8.44 (d, *J* = 15.1 Hz, 3H), 8.16 (d, *J* = 8.5 Hz, 1H), 7.76 (ddd, *J* = 16.2, 8.7, 4.0 Hz, 3H), 7.61–7.48 (m, 7H), 7.48–7.41 (m, 1H), 7.31 (t, *J* = 7.4 Hz, 1H), 7.09 (dd, *J* = 7.5, 3.7 Hz, 4H), 4.46 (s, 2H, –CH_2_–), 3.58(s, N–CH_2_Py, 4H). ^13^C NMR (101 MHz, CDCl_3_, δ ppm): 155.86; 149.73; 148.75; 148.64; 146.51; 141.62; 139.75; 136.07; 134.23; 130.37; 130.35; 129.23; 129.07, 128.99; 128.53; 126.76; 125.38; 124.38; 123.52; 123.46; 122.00; 120.89; 117.62; 59.93 (–N**C**H_2_Py); 50.67 [–**C**H_2_N(CH_2_Py)_2_]. Anal. Calcd for C_35_H_28_N_6_: C 78.92%; H 5.30%; N 15.78%. Found C 78.68%; H 5.26%; N 15.54%. The NMR spectra can be found in the ESI† file (Figures S7 and S8).

### 3.2. Materials, UV–Vis, and Fluorescence Measurements

The solvents *n*-hexane (HEX), diethyl ether (DEE), ethyl acetate (EA) tetrahydrofuran (THF), methanol (MeOH), and acetonitrile (ACN) were received from commercial vendors and used as received. All the solvents were of spectroscopic grade and did not show any traces in the absorption or fluorescence spectra. The concentration of the PQPc sample was equal to 1.73 × 10^−5^ M. Nitrates(V) of zinc, cadmium, copper, magnesium, cobalt, nickel, calcium, and lead were used to prepare the ion solution. The salts were used as received. Subsequent portions of ion solutions were added to the acetonitrile solution of PQPc and the reference, absorption, and fluorescence measurements were performed. Measurements were stopped when a non-changing fluorescence intensity was obtained.

The absorption spectra were recorded using the UV-3600 Shimadzu Spectrophotometer and the emission spectra were measured using the Hitachi F7000 Spectrofluorometer. For all fluorescence measurements, the excitation wavelength was 380 nm. A solution of quinine sulfate in 0.05 M of sulfuric acid was used to calibrate the sensitivity of the photomultiplayer. Fluorescence quantum yield was calculated using quinine sulfate as a standard, where the quantum yield of the standard was 0.577 [47].

In order to determine the detection limit of the chosen ion, a range of changing fluorescence intensities was selected and fluorescence measurements were performed. Ion solution with a concentration from 1 × 10^−6^ to 1 × 10^−4^ M was added to PQPc in acetonitrile, increasing the concentration by 1 × 10^−6^ M in subsequent portions. Job’s method was used to determine the stoichiometry of the complex. For this purpose, a series of PQPc and Zn^2+^ solutions were prepared with a total PQPc + Zn^2+^ concentration of 1.7 × 10^−5^ M and a variable molar ratio of Zn^2+^ to PQPc. Job’s plot [48] was presented as the difference I_fl_ − I_fl0_ in relation to the molar fraction of Zn^2+^.

### 3.3. Biological Methods

#### 3.3.1. Cytotoxicity Activity (MTS Assay)

Chinese Hamster Ovary (CHO-K1) cells were cultured at 37 °C in a humidified 5% CO_2_ atmosphere in plastic dishes in F-12K medium (Sigma Aldrich Chemicals, Rockville, MD, USA) supplemented with 10% fetal bovine serum (Invitrogen, Carlsbad, CA, USA), 2 mM of L-glutamine (Sigma Aldrich Chemicals, USA), and antibiotics (100 units/mL of penicillin and 100 µg/mL of streptomycin (Invitrogen, USA)) at 37 °C.

Cytotoxic properties of the Zn(II) ions and PQPc sensor were measured using an MTS Cell Proliferation Assay Kit (Abcam, Cambridge, United Kingdom) in accordance with the manufacturer’s instructions. Cells were seeded into a 96-well plate and incubated with the Zn(II) ions in the concentrations of 0.001, 0.003, 0.005, 0.01, and 0.1 mM for 24 h at 37 °C in a humidified atmosphere of 5% CO_2_. After incubation, a solution of MTS (3-(4,5-dimethylthiazol-2-yl)-5-(3-carboxymethoxyphenyl)-2-(4-sulfophenyl)-2H-tetrazolium) was added to each well and incubated at 37 °C for 4 h. The measurement of the absorbance of the solution related to the number of live cells was conducted on a TECAN Spark Microplate Reader (TECAN, Männedorf, Switzerland) at 490 nm. All samples were tested in three independent experiments. The results were normalized to the control.

#### 3.3.2. Detection of Zn in Cells Using a Sensor

Cells were seeded into a 96-well black plate and incubated with the Zn(II) ions in the concentrations of 0.001, 0.003, 0.005, 0.01, and 0.1 mM for 24 h at 37 °C in a humidified atmosphere of 5% CO_2_. After incubation, the Zn(II) medium was removed and the cells were washed twice with PBS. The cells were flooded with fresh medium. A sensor was added and fluorescence with an emission wavelength of 380 nm and an excitation wavelength of 495 nm was measured in a TECAN Infinite 200 PRO microplate reader (TECAN Group Ltd., Mannedorf, Switzerland). Confocal fluorescence images were acquired with the Nikon A1R confocal microscope equipped with the compact FLIM and FCS Pico Quant modules.

### 3.4. Computational Details

Density functional theory (DFT) [49,50] computations were performed using Gaussian16 [51]. With the cc-pVDZ standard basis set, the ground-state structures of molecules were optimized using the B3LYP method [52,53]. By computing the Hessian matrix and confirming that there were no imaginary frequencies, the real energy minima of the optimized structures were verified. The vertical excited-state energies of the dye and analyte–dye complex along with the optimization of their structures in the excited state were determined using the TD-B3LYP/cc-pVDZ method. The polarity effect induced by acetonitrile was simulated using the Polarizable Continuum Model (PCM) [54,55].

## 4. Conclusions

In summary, a new pyrazoloquinoline derivative with a dipicolylamine chelator was synthesized as a sensitive and selective fluorescence sensor for Zn^2+^ ion recognition. The sensor, PQPc, exhibited a clear solvent-polarity dependence of fluorescence quantum yields. The observed low fluorescence quantum yield in polar acetonitrile was explained in the context of the PET process activation. Taking advantage of the phenomenon, we tested our dye as an optical sensor employing several fluorometric experiments: bivalent metal ion selectivity experiment, bivalent metal ion competition studies, and fluorescence titration analysis. The fluorometric titration experiment showed that the greatest increase in fluorescence quantum yield of the sensor (compared with the other bivalent cations under the study) was only observed in the presence of Zn^2+^ ions. The stoichiometry of the Zn^2+^ and PQPc complex was established to be 1:1, with the binding constant equal to 859 M^−1^. The estimated detection limit of Zn^2+^ was found to be 1.93 × 10^−7^ M, which corresponds to the concentration being approximately 90 times lower than the ligand concentration. The sensor was selective toward the Zn^2+^ ion in the presence of Pb^2+^, Ca^2+^, Cd^2+^, and Mg^2+^ ions. The addition of Co^2+^, Cu^2+^, and Ni^2+^ quenched fluorescence, which allowed us to build an INHIBIT logic gate with Zn^2+^ and Cu^2+^. Biological studies showed that in the future, PQPc may be applied to detect zinc ions in eukaryotic cells.

## Figures and Tables

**Table 1:** The value of absorption maximum, λ_abs_ [nm], the molar absorption coefficient, fluorescence maximum, λ_fl_ [nm], and fluorescence quantum yield, $\phi_{\mathrm{fl}}$, for PQPc as a function of solvent polarity function, $f\left(\varepsilon ,n\right)=\frac{\varepsilon -1}{2\varepsilon +1}-\frac{n^{2}-1}{2n^{2}+1}$.

Solvent	f(ε,n)	λ_abs_ [nm]	λ_fl_ [nm]	$\phi_{fl}$ [%]
HEX	0	395	454	12.87
DEE	0.095	393	466	2.03
EA	0.202	392	469	1.40
THF	0.209	395	476	1.20
MeOH	0.309	394	476	0.99
ACN	0.305	396	483	0.75

## Data Availability

Data are contained within the article or Supplementary Materials.

## Supplementary Materials

The following supporting information can be downloaded at: https://www.mdpi.com/article/10.3390/molecules29040823/s1, Figure S1: Experimental and DFT-predicted spectra of the PQPc and of the PQPc+Zn^2+^ in acetonitrile, Figure S2: Experimental and DFT-predicted emission spectra of the PQPc and of the PQPc+Zn^2+^ in acetonitrile, Figure S3: Comparison of the HOMO-1, HOMO, and LUMO orbitals calculated for the PQPc complex in the ground-state geometry with the same type of orbitals in the excited-state geometry, Figure S4: Comparison of the HOMO-1, HOMO, and LUMO orbitals calculated for the PQPc+Zn^2+^ complex in the ground-state geometry with the same type of orbitals in the excited-state geometry, Figure S5: The diagram of selectivity of PQPc toward Zn^2+^ ion in the presence of other cation, Figure S6: Fluorescence enhancement of PQPc in cells treated with zinc(II) ions (λ_ex_=380 nm, λ_em_=495 nm), Figure S7: ^1^H NMR recorded for the investigated sensor, Figure S8: ^13^C NMR recorded for the investigated sensor; Cartesian coordinates for the optimized structures of the sensor and the complex with Zn^2+^ (both in ground and excited state).
