# Supplementary material for: Fluorescent Sensor Based on 1H-Pyrazolo[3,4-b]quinoline Derivative for Detecting Zn2+ Cations
Source: Molecules. 2024 Feb 10;29(4):823. doi: 10.3390/molecules29040823 (PMC10891916; doi:10.3390/molecules29040823)
Supplement: Supplementary file 1 [file molecules-29-00823-s001.zip › molecules-2768683-supplementary.pdf]

Supplementary Information

Fluorescent Sensor Based on 1*H*-Pyrazolo[3,4-*b*]quinoline Derivative for Detecting Zn^{2+} Cations

Anna Kolbus [1], Tomasz Uchacz [2,*], Andrzej Danel [3], Katarzyna Gałczyńska [4], Paulina Moskwa [1] and Przemysław Kolek [5]

[1] Institute of Chemistry, The Jan Kochanowski University, Uniwersytecka 7 St., 25-406 Kielce, Poland; anna.kolbus@ujk.edu.pl (A.K.); paulinam1999@tlen.pl (P.M.)
[2] Faculty of Chemistry, Jagiellonian University, Gronostajowa 2 St., 30-387 Kraków, Poland
[3] Faculty of Materials Engineering and Physics, Cracow University of Technology, Podchorążych St.1, 30-348 Kraków, Poland; rrdanela@cyf-kr.edu.pl
[4] Institute of Biology, The Jan Kochanowski University, Uniwersytecka 7 St., 25-406 Kielce, Poland; kgalczynska@ujk.edu.pl
[5] Institute of Physics, University of Rzeszów, 1 Pigonia St., 35-310 Rzeszów, Poland; pkolek@ur.edu.pl
* Correspondence: uchacz@chemia.uj.edu.pl

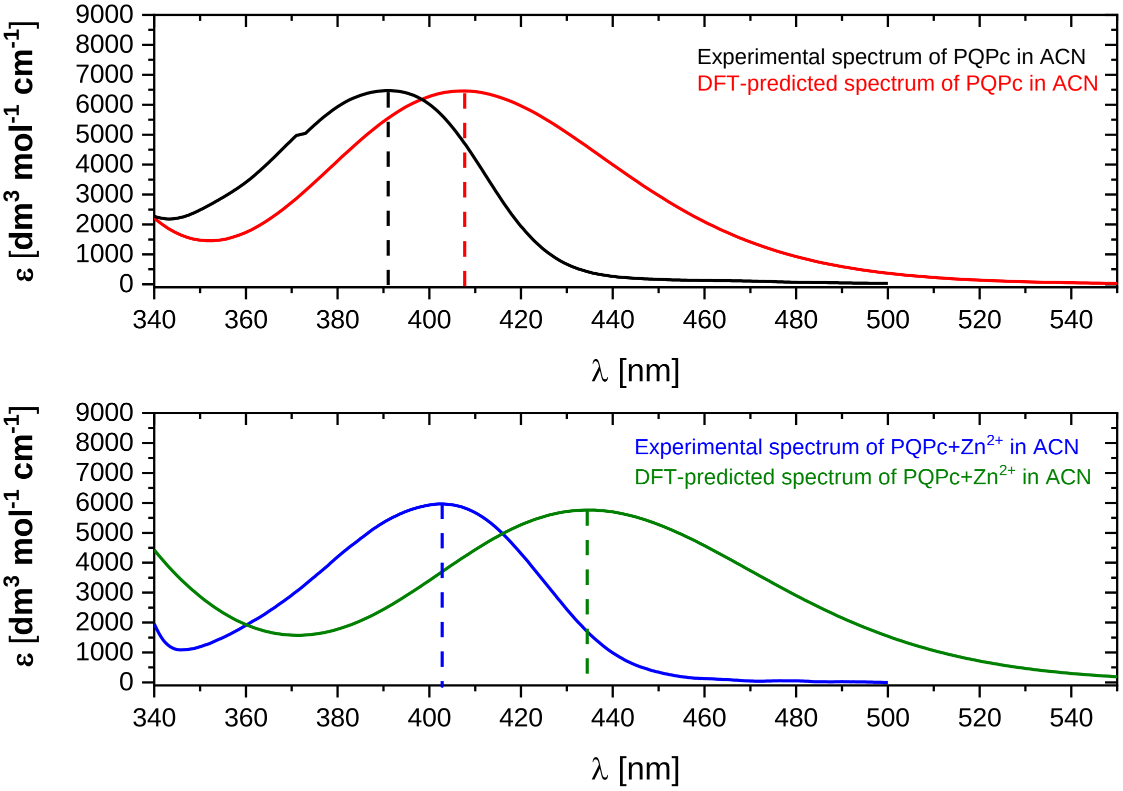

Figure S1. Experimental and DFT-predicted spectra of the PQPc and of the PQPc+Zn^{2+} in acetonitrile.

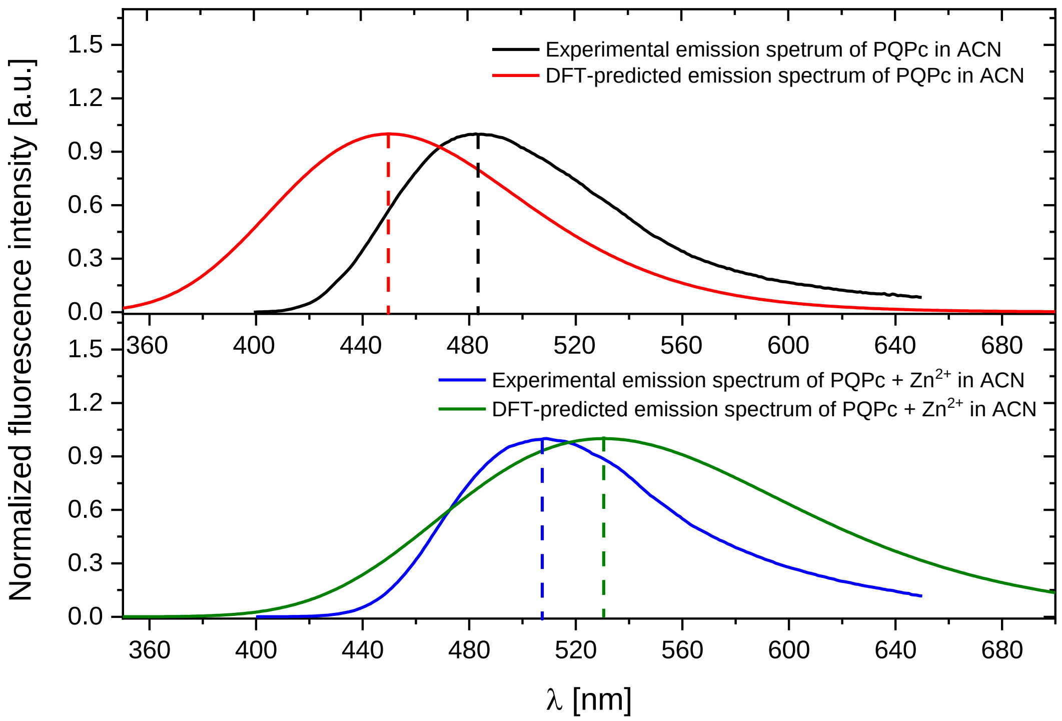

Figure S2. Experimental and DFT-predicted emission spectra of the PQPc and of the PQPc+Zn^{2+} in acetonitrile.

	Ground state geometry	Excited state geometry
LUMO	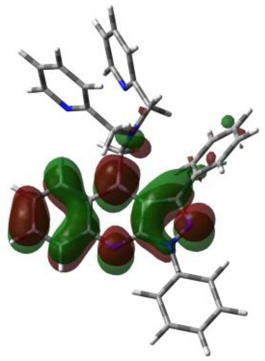	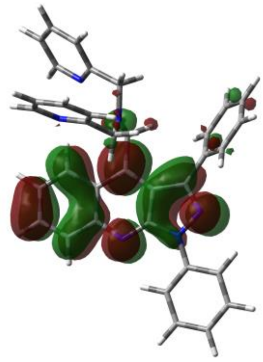
HOMO	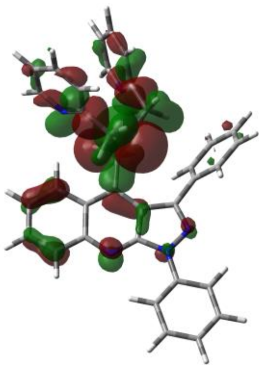	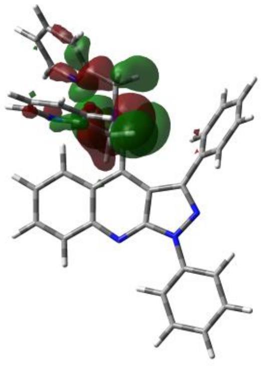
HOMO-1	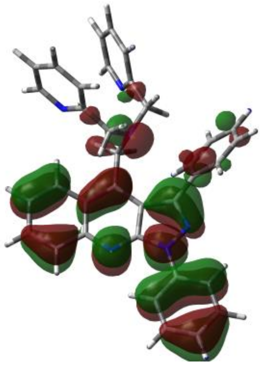	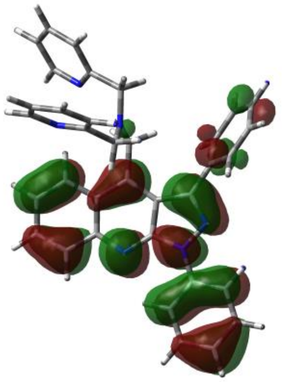

Figure S3. Comparison of the HOMO-1, HOMO, and LUMO orbitals calculated for the PQPc complex in the ground-state geometry with the same type of orbitals in the excited-state geometry.

	Ground state geometry	Excited state geometry
LUMO	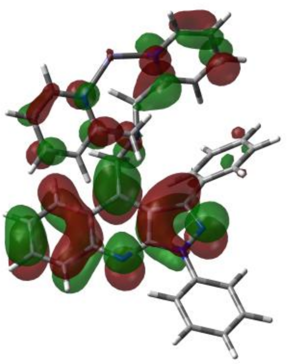	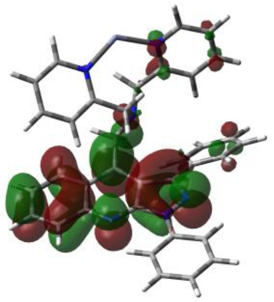
HOMO	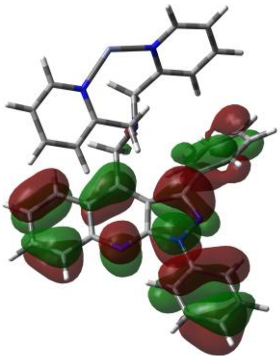	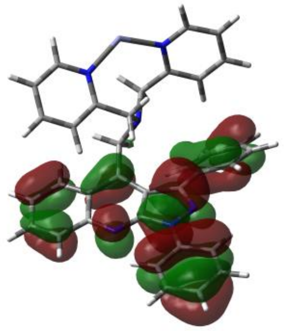
HOMO-1	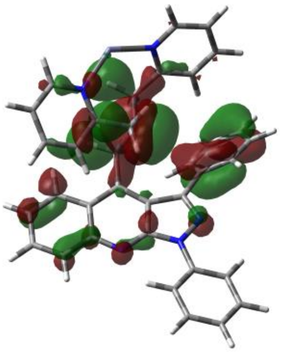	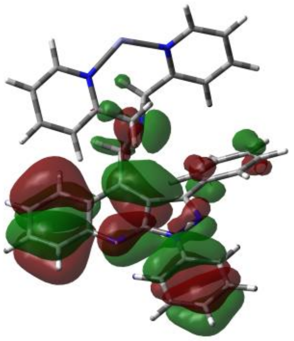

Figure S4. Comparison of the HOMO-1, HOMO, and LUMO orbitals calculated for the PQPc+Zn^{2+} complex in the ground-state geometry with the same type of orbitals in the excited-state geometry.

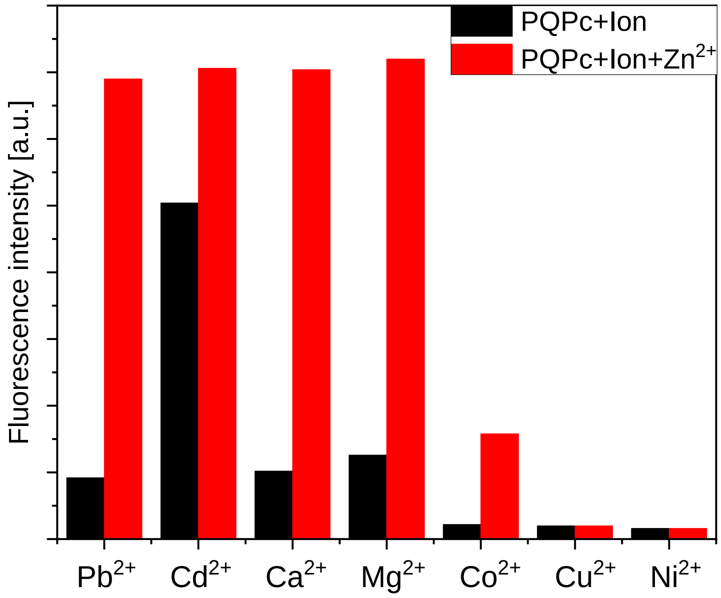

Figure S5. The diagram of selectivity of PQPc toward Zn^{2+} ion in the presence of other cation.

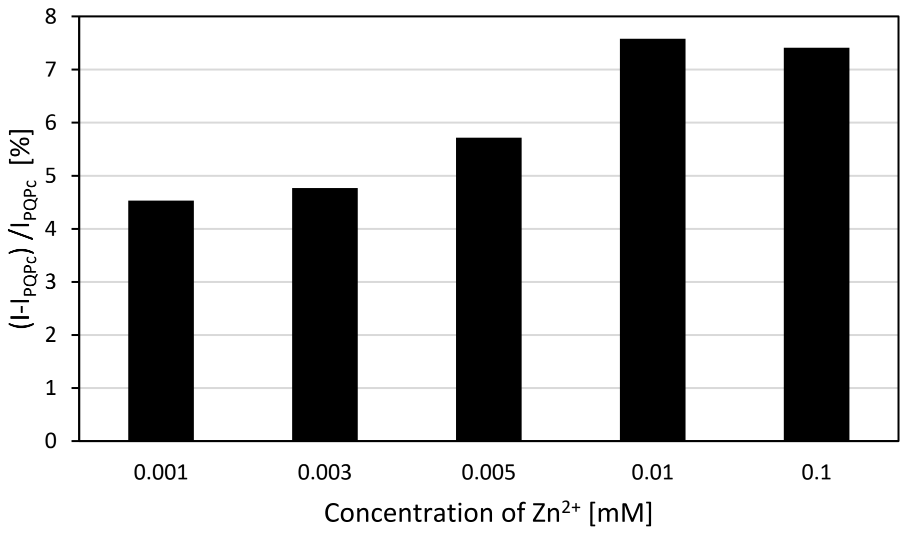

Figure S6. Fluorescence enhancement of PQPc in cells treated with zinc(II) ions (λ_{ex}=380 nm, λ_{em}=495 nm).

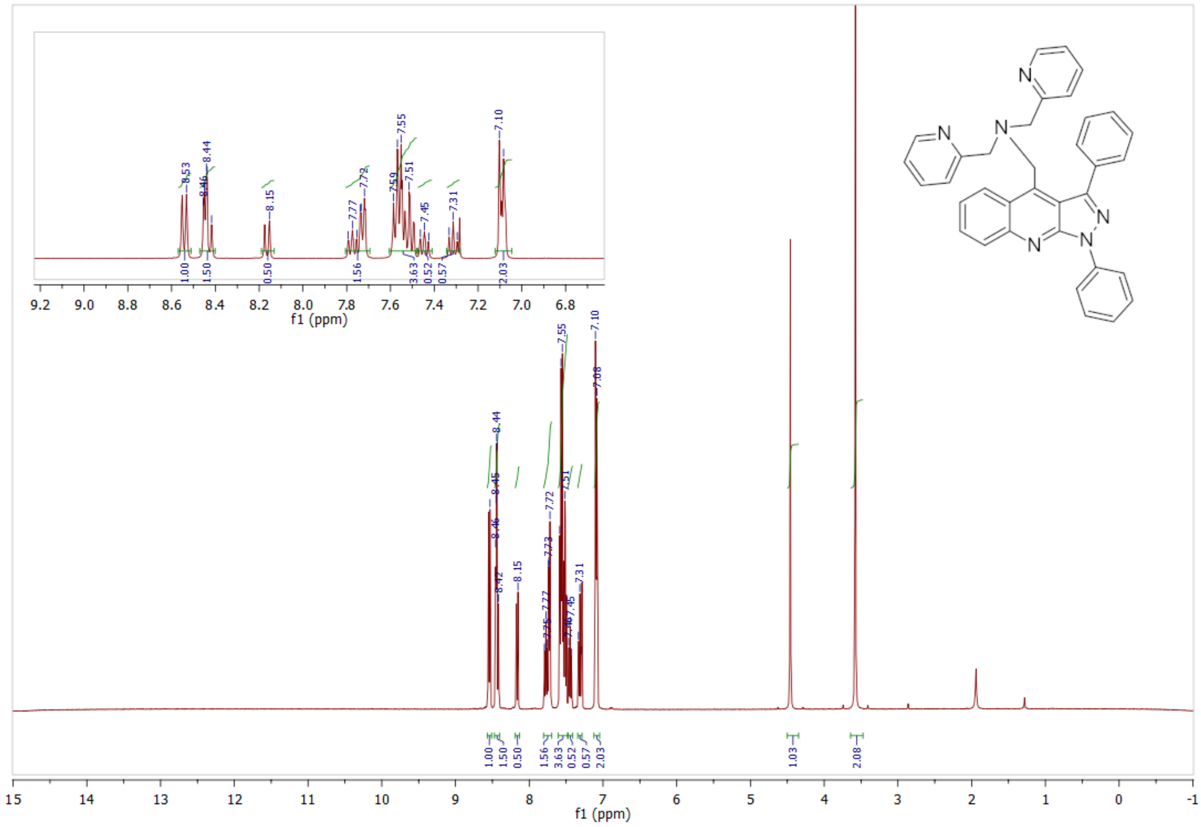

Figure S7. ^{1}H NMR recorded for the investigated sensor.

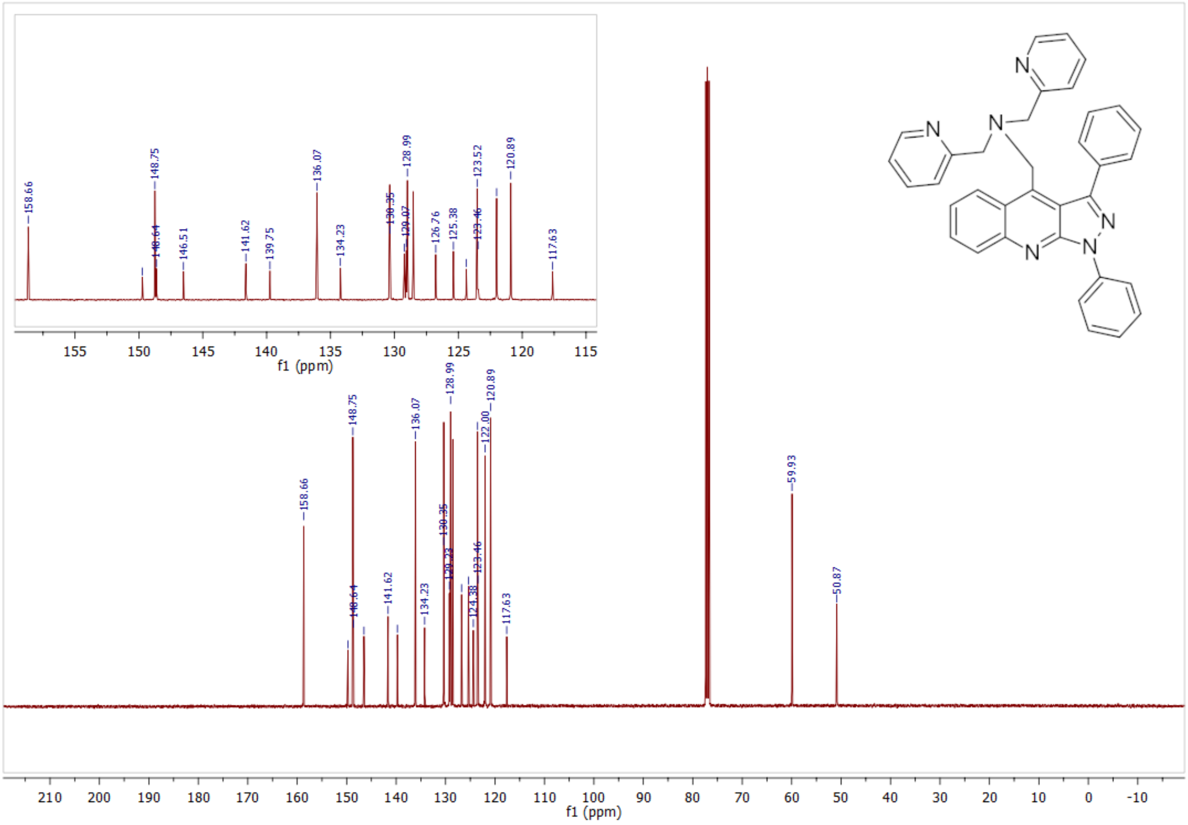

Figure S8. ^{13}C NMR recorded for the investigated sensor.

Optimized (B3LYP/cc-pVDZ) ground-state geometries of PQPc in vacuum.

Row	Highlight	Display	Tag	Symbol	X	Y	Z
1	No	Show	1	C	-0.3290940	-4.3335550	-1.4076370
2	No	Show	2	C	0.9749810	-4.8927220	-1.3344580
3	No	Show	3	C	2.0513800	-4.0937680	-1.0333060
4	No	Show	4	C	1.8870500	-2.6948450	-0.8055550
5	No	Show	5	C	0.5664070	-2.1096540	-0.9122590
6	No	Show	6	C	-0.5277520	-2.9881130	-1.2010360
7	No	Show	7	N	2.9968870	-1.9865550	-0.4750080
8	No	Show	8	C	2.8052660	-0.6939800	-0.2963630
9	No	Show	9	C	1.5683030	0.0212570	-0.4393680
10	No	Show	10	C	0.4038050	-0.7022060	-0.7180620
11	No	Show	11	N	3.7639410	0.2384070	0.0716680
12	No	Show	12	N	3.2119620	1.4850840	0.1374560
13	No	Show	13	C	1.9325440	1.3992340	-0.1704490
14	No	Show	14	C	1.1497510	2.6570370	-0.2248410
15	No	Show	15	C	5.1532090	0.0880950	0.3403020
16	No	Show	16	C	0.5044870	3.0579700	-1.4067090
17	No	Show	17	C	-0.1728850	4.2786880	-1.4689650
18	No	Show	18	C	-0.2117170	5.1180710	-0.3515690
19	No	Show	19	C	0.4384410	4.7330040	0.8255440
20	No	Show	20	C	1.1180190	3.5143810	0.8872560
21	No	Show	21	C	5.7729180	-1.1713690	0.2978790
22	No	Show	22	C	7.1391300	-1.2730270	0.5727660
23	No	Show	23	C	7.8970860	-0.1435630	0.8893570
24	No	Show	24	C	7.2705190	1.1061380	0.9287050
25	No	Show	25	C	5.9085610	1.2303800	0.6569390
26	No	Show	26	C	-0.9618860	-0.0309580	-0.7773280
27	No	Show	27	N	-1.4226520	0.3783270	0.5556670
28	No	Show	28	C	-1.7066160	-0.6709250	1.5240370
29	No	Show	29	C	-2.2784760	1.5613750	0.6109730
30	No	Show	30	C	-3.0583960	-1.3797270	1.4618040
31	No	Show	31	C	-3.6446180	1.4932890	-0.0654690
32	No	Show	32	C	-3.8514540	-1.5029220	2.6129770
33	No	Show	33	C	-5.0710400	-2.1765280	2.5369650
34	No	Show	34	C	-5.4708060	-2.6985900	1.3063510
35	No	Show	35	C	-4.6178300	-2.5260100	0.2129440
36	No	Show	36	N	-3.4396970	-1.8944720	0.2779190
37	No	Show	37	N	-3.6348360	1.4244890	-1.4098630
38	No	Show	38	C	-4.8098220	1.3790560	-2.0480570
39	No	Show	39	C	-6.0488710	1.4176780	-1.4023680
40	No	Show	40	C	-6.0588630	1.5031910	-0.0094920
41	No	Show	41	C	-4.8392870	1.5392590	0.6676610
42	No	Show	42	H	-1.1832020	-4.9793490	-1.6228740
43	No	Show	43	H	1.1178600	-5.9621010	-1.5069220
44	No	Show	44	H	3.0626970	-4.4956730	-0.9533250
45	No	Show	45	H	-1.5416370	-2.5904130	-1.2211010
46	No	Show	46	H	0.5561230	2.4214310	-2.2925660
47	No	Show	47	H	-0.6670000	4.5757560	-2.3965720
48	No	Show	48	H	-0.7422800	6.0717710	-0.3996910

49	No	Show	49	H	0.4188130	5.3868740	1.7006790
50	No	Show	50	H	1.6323040	3.2136590	1.8014800
51	No	Show	51	H	5.1833790	-2.0500430	0.0495510
52	No	Show	52	H	7.6120180	-2.2573390	0.5367340
53	No	Show	53	H	8.9642800	-0.2344290	1.1025390
54	No	Show	54	H	7.8469000	2.0015870	1.1730830
55	No	Show	55	H	5.4151190	2.1997410	0.6836680
56	No	Show	56	H	-1.6874730	-0.6663490	-1.3024780
57	No	Show	57	H	-0.8854410	0.8861530	-1.3735490
58	No	Show	58	H	-1.5954670	-0.2426790	2.5360460
59	No	Show	59	H	-0.9183640	-1.4356560	1.4257160
60	No	Show	60	H	-2.4143860	1.8196610	1.6742310
61	No	Show	61	H	-1.7275680	2.3920550	0.1434080
62	No	Show	62	H	-3.5104210	-1.0734710	3.5579650
63	No	Show	63	H	-5.7008690	-2.2876230	3.4231300
64	No	Show	64	H	-6.4176190	-3.2296470	1.1913710
65	No	Show	65	H	-4.8948400	-2.9225150	-0.7697790
66	No	Show	66	H	-4.7596400	1.3178550	-3.1409310
67	No	Show	67	H	-6.9755240	1.3867750	-1.9790010
68	No	Show	68	H	-7.0016950	1.5415580	0.5417630
69	No	Show	69	H	-4.8099720	1.6015890	1.7575200

Optimized (B3LYP/cc-pVDZ) ground-state geometries of PQPc in ACN.

Row	Highlight	Display	Tag	Symbol	X	Y	Z
1	No	Show	1	C	-0.3068820	-4.3554100	-1.3429330
2	No	Show	2	C	0.9998800	-4.9097000	-1.2727390
3	No	Show	3	C	2.0760060	-4.1042270	-0.9832780
4	No	Show	4	C	1.9081610	-2.7040110	-0.7650980
5	No	Show	5	C	0.5847380	-2.1245640	-0.8663030
6	No	Show	6	C	-0.5082280	-3.0084830	-1.1436660
7	No	Show	7	N	3.0192780	-1.9865450	-0.4515570
8	No	Show	8	C	2.8223080	-0.6929660	-0.2814800
9	No	Show	9	C	1.5800830	0.0134350	-0.4152960
10	No	Show	10	C	0.4163130	-0.7164160	-0.6806480
11	No	Show	11	N	3.7809740	0.2482520	0.0647700
12	No	Show	12	N	3.2208060	1.4944700	0.1256930
13	No	Show	13	C	1.9378830	1.3965190	-0.1635520
14	No	Show	14	C	1.1435290	2.6482040	-0.2217250
15	No	Show	15	C	5.1714300	0.1073570	0.3275180
16	No	Show	16	C	0.5169380	3.0488210	-1.4145160
17	No	Show	17	C	-0.1699630	4.2645220	-1.4817510
18	No	Show	18	C	-0.2379360	5.0966310	-0.3590980
19	No	Show	19	C	0.3913100	4.7099000	0.8293400
20	No	Show	20	C	1.0816260	3.4964320	0.8965970
21	No	Show	21	C	5.8205270	-1.1313840	0.1888180
22	No	Show	22	C	7.1894620	-1.2259100	0.4569720
23	No	Show	23	C	7.9240750	-0.1075200	0.8600170
24	No	Show	24	C	7.2700980	1.1221890	0.9954090
25	No	Show	25	C	5.9043510	1.2370700	0.7338870

26	No	Show	26	C	-0.9525090	-0.0496500	-0.7428680
27	No	Show	27	N	-1.4280970	0.3421200	0.5891860
28	No	Show	28	C	-1.7535710	-0.7238060	1.5314750
29	No	Show	29	C	-2.2684750	1.5385520	0.6523690
30	No	Show	30	C	-3.1104640	-1.4166470	1.4203780
31	No	Show	31	C	-3.6276400	1.5089600	-0.0411980
32	No	Show	32	C	-3.9410520	-1.5299920	2.5455940
33	No	Show	33	C	-5.1637620	-2.1936430	2.4318020
34	No	Show	34	C	-5.5281480	-2.7167980	1.1898920
35	No	Show	35	C	-4.6400190	-2.5548060	0.1232720
36	No	Show	36	N	-3.4575050	-1.9312280	0.2244430
37	No	Show	37	N	-3.6096590	1.4807110	-1.3885890
38	No	Show	38	C	-4.7837020	1.4725420	-2.0356000
39	No	Show	39	C	-6.0254220	1.5113720	-1.3954540
40	No	Show	40	C	-6.0427540	1.5572670	-0.0000400
41	No	Show	41	C	-4.8265100	1.5539220	0.6853650
42	No	Show	42	H	-1.1592090	-5.0060040	-1.5495820
43	No	Show	43	H	1.1453920	-5.9795510	-1.4380900
44	No	Show	44	H	3.0876110	-4.5069320	-0.9071850
45	No	Show	45	H	-1.5238930	-2.6142150	-1.1590350
46	No	Show	46	H	0.5885590	2.4172080	-2.3025690
47	No	Show	47	H	-0.6479510	4.5642060	-2.4168630
48	No	Show	48	H	-0.7761850	6.0455600	-0.4115370
49	No	Show	49	H	0.3460760	5.3563220	1.7086790
50	No	Show	50	H	1.5745850	3.1951310	1.8226160
51	No	Show	51	H	5.2510730	-2.0028190	-0.1231250
52	No	Show	52	H	7.6827490	-2.1942560	0.3453670
53	No	Show	53	H	8.9929410	-0.1915410	1.0664710
54	No	Show	54	H	7.8264690	2.0081750	1.3098040
55	No	Show	55	H	5.3947480	2.1927200	0.8378250
56	No	Show	56	H	-1.6664860	-0.6854100	-1.2836660
57	No	Show	57	H	-0.8686940	0.8728240	-1.3292910
58	No	Show	58	H	-1.6686180	-0.3126840	2.5516630
59	No	Show	59	H	-0.9696930	-1.4943210	1.4439870
60	No	Show	60	H	-2.4165280	1.7777140	1.7176250
61	No	Show	61	H	-1.6962870	2.3681390	0.2086610
62	No	Show	62	H	-3.6290180	-1.0987480	3.4992050
63	No	Show	63	H	-5.8230090	-2.2952970	3.2969020
64	No	Show	64	H	-6.4749850	-3.2401290	1.0459800
65	No	Show	65	H	-4.8901660	-2.9538040	-0.8654520
66	No	Show	66	H	-4.7305680	1.4438140	-3.1294580
67	No	Show	67	H	-6.9484010	1.5113100	-1.9781050
68	No	Show	68	H	-6.9879390	1.5956420	0.5464260
69	No	Show	69	H	-4.8032450	1.5859020	1.7763520

Optimized (B3LYP/cc-pVDZ) excited-state geometries of PQPc in ACN.

Row	Highlight	Display	Tag	Symbol	X	Y	Z
1	No	Show	1	C	-0.7647670	-3.6153310	-2.2351220
2	No	Show	2	C	0.4329890	-4.3390500	-2.1416020
3	No	Show	3	C	1.5757030	-3.7127590	-1.6404790

4	No	Show	4	C	1.5683160	-2.3604540	-1.2321510
5	No	Show	5	C	0.3415160	-1.5965570	-1.3555360
6	No	Show	6	C	-0.8096580	-2.2768730	-1.8448600
7	No	Show	7	N	2.7373080	-1.8373650	-0.7149950
8	No	Show	8	C	2.6808920	-0.5552880	-0.3660840
9	No	Show	9	C	1.5690420	0.3340560	-0.4866270
10	No	Show	10	C	0.3381310	-0.2129680	-0.9705160
11	No	Show	11	N	3.7106800	0.1813690	0.2107060
12	No	Show	12	N	3.3211850	1.4800500	0.4501620
13	No	Show	13	C	2.0527790	1.5783270	0.0246510
14	No	Show	14	C	1.3891040	2.8974890	0.1565370
15	No	Show	15	C	5.0318360	-0.1992780	0.5438770
16	No	Show	16	C	0.8016690	3.5461880	-0.9472370
17	No	Show	17	C	0.2056630	4.8043550	-0.8082420
18	No	Show	18	C	0.1974480	5.4458430	0.4346780
19	No	Show	19	C	0.7979530	4.8227670	1.5352040
20	No	Show	20	C	1.3888600	3.5641150	1.3967850
21	No	Show	21	C	5.5159980	-1.4959970	0.2868640
22	No	Show	22	C	6.8289040	-1.8278560	0.6345150
23	No	Show	23	C	7.6765880	-0.8925110	1.2354770
24	No	Show	24	C	7.1907250	0.3958080	1.4879220
25	No	Show	25	C	5.8834430	0.7464840	1.1489530
26	No	Show	26	C	-0.9035880	0.6099360	-1.0916510
27	No	Show	27	N	-1.8396930	0.5707600	0.0384760
28	No	Show	28	C	-1.5041630	-0.0579590	1.3052850
29	No	Show	29	C	-3.0607980	1.3594810	-0.0779390
30	No	Show	30	C	-2.6559200	-0.8174270	1.9306500
31	No	Show	31	C	-4.1593900	0.7042650	-0.9231240
32	No	Show	32	C	-2.9823610	-0.6450780	3.2799710
33	No	Show	33	C	-4.0019520	-1.4306810	3.8238100
34	No	Show	34	C	-4.6591860	-2.3427170	2.9975420
35	No	Show	35	C	-4.2670380	-2.4286130	1.6579430
36	No	Show	36	N	-3.2834070	-1.6914480	1.1252460
37	No	Show	37	N	-3.8068750	0.2030860	-2.1101060
38	No	Show	38	C	-4.7678710	-0.3112290	-2.8922430
39	No	Show	39	C	-6.1162080	-0.3310780	-2.5308760
40	No	Show	40	C	-6.4773680	0.2017000	-1.2919570
41	No	Show	41	C	-5.4814270	0.7251250	-0.4653240
42	No	Show	42	H	-1.6713280	-4.0974640	-2.6093790
43	No	Show	43	H	0.4726450	-5.3869090	-2.4481920
44	No	Show	44	H	2.5165350	-4.2598160	-1.5427100
45	No	Show	45	H	-1.7613620	-1.7479590	-1.9057180
46	No	Show	46	H	0.8444230	3.0766930	-1.9330360
47	No	Show	47	H	-0.2402940	5.2901880	-1.6792370
48	No	Show	48	H	-0.2667630	6.4285200	0.5437230
49	No	Show	49	H	0.8029570	5.3189020	2.5087370
50	No	Show	50	H	1.8565030	3.0793930	2.2557870
51	No	Show	51	H	4.8530870	-2.2202820	-0.1799420
52	No	Show	52	H	7.1893370	-2.8388360	0.4281740
53	No	Show	53	H	8.7005760	-1.1616560	1.5031110
54	No	Show	54	H	7.8363020	1.1430430	1.9561470

55	No	Show	55	H	5.5032290	1.7474490	1.3416020
56	No	Show	56	H	-1.5362520	0.3499900	-1.9616360
57	No	Show	57	H	-0.6791830	1.6808400	-1.2255840
58	No	Show	58	H	-1.1532100	0.7465930	1.9751750
59	No	Show	59	H	-0.6520450	-0.7233550	1.1171710
60	No	Show	60	H	-3.4405950	1.5706880	0.9269210
61	No	Show	61	H	-2.7804470	2.3117050	-0.5519050
62	No	Show	62	H	-2.4491860	0.0840420	3.8923000
63	No	Show	63	H	-4.2788620	-1.3263120	4.8745760
64	No	Show	64	H	-5.4616380	-2.9768800	3.3774410
65	No	Show	65	H	-4.7658420	-3.1273120	0.9794850
66	No	Show	66	H	-4.4407380	-0.7208590	-3.8520190
67	No	Show	67	H	-6.8606800	-0.7552830	-3.2060620
68	No	Show	68	H	-7.5197540	0.2062900	-0.9678280
69	No	Show	69	H	-5.7256710	1.1403970	0.5133340

Optimized (B3LYP/cc-pVDZ) ground-state geometries of PQPc+Zn^{2+} in vacuum.

Row	Highlight	Display	Tag	Symbol	X	Y	Z
1	No	Show	1	C	0.8963610	4.6288840	2.0783640
2	No	Show	2	C	2.1401800	4.9884150	1.4922920
3	No	Show	3	C	2.9010510	4.0340030	0.8612520
4	No	Show	4	C	2.4648870	2.6768040	0.7899210
5	No	Show	5	C	1.1825000	2.3030060	1.3570790
6	No	Show	6	C	0.4289320	3.3352280	2.0036520
7	No	Show	7	N	3.2849000	1.7970530	0.1666820
8	No	Show	8	C	2.9005710	0.5401590	0.1950830
9	No	Show	9	C	1.6886060	0.0151200	0.7696960
10	No	Show	10	C	0.7570830	0.9387570	1.2630820
11	No	Show	11	N	3.5913550	-0.5404260	-0.3363880
12	No	Show	12	N	2.9343920	-1.6897450	-0.0723200
13	No	Show	13	C	1.8150290	-1.4191800	0.5869150
14	No	Show	14	C	1.0485510	-2.5732750	1.1006140
15	No	Show	15	C	4.8639070	-0.5956560	-0.9802990
16	No	Show	16	C	0.5136330	-2.5840510	2.4034670
17	No	Show	17	C	-0.0843120	-3.7380710	2.9224350
18	No	Show	18	C	-0.1409850	-4.9076310	2.1563570
19	No	Show	19	C	0.4049900	-4.9138240	0.8658700
20	No	Show	20	C	0.9889020	-3.7588960	0.3402750
21	No	Show	21	C	5.4328390	0.5542630	-1.5537770
22	No	Show	22	C	6.6739900	0.4550030	-2.1820850
23	No	Show	23	C	7.3467850	-0.7697800	-2.2477130
24	No	Show	24	C	6.7687270	-1.9095940	-1.6748210
25	No	Show	25	C	5.5321860	-1.8318230	-1.0400220
26	No	Show	26	C	-0.6539740	0.5303720	1.6342200
27	No	Show	27	N	-1.3945360	-0.0884570	0.5029720
28	No	Show	28	C	-1.4405160	0.6645320	-0.7564530
29	No	Show	29	C	-2.6023420	-0.7572150	0.9271670
30	No	Show	30	C	-2.1359730	2.0308010	-0.7877940
31	No	Show	31	C	-3.1179670	-1.8309790	-0.0192440
32	No	Show	32	C	-1.4004700	3.2144500	-0.8521920
33	No	Show	33	C	-2.0476980	4.4509080	-0.9211120
34	No	Show	34	C	-3.4437280	4.4959570	-0.9152000
35	No	Show	35	C	-4.1425460	3.3001560	-0.8534900
36	No	Show	36	N	-3.5075870	2.0995630	-0.7982040
37	No	Show	37	N	-4.1653500	-1.4965710	-0.8264220
38	No	Show	38	C	-4.7304070	-2.4099490	-1.6542920
39	No	Show	39	C	-4.2566380	-3.7131210	-1.7073400
40	No	Show	40	C	-3.1682640	-4.0680750	-0.9032420
41	No	Show	41	C	-2.5912370	-3.1183500	-0.0550510
42	No	Show	42	H	0.3149680	5.3833650	2.6122390
43	No	Show	43	H	2.4959020	6.0179260	1.5623720
44	No	Show	44	H	3.8693410	4.2699100	0.4179600
45	No	Show	45	H	-0.5123120	3.0972680	2.4995490
46	No	Show	46	H	0.6459490	-1.7159140	3.0523710
47	No	Show	47	H	-0.4640380	-3.7367360	3.9465070
48	No	Show	48	H	-0.5790530	-5.8164040	2.5741550

49	No	Show	49	H	0.3966680	-5.8317090	0.2733370
50	No	Show	50	H	1.4363790	-3.7717420	-0.6545600
51	No	Show	51	H	4.9150740	1.5079320	-1.4989660
52	No	Show	52	H	7.1178150	1.3469590	-2.6278850
53	No	Show	53	H	8.3182360	-0.8363870	-2.7406390
54	No	Show	54	H	7.2898940	-2.8676810	-1.7155570
55	No	Show	55	H	5.0792460	-2.7106880	-0.5851760
56	No	Show	56	H	-1.2118960	1.3888850	2.0423720
57	No	Show	57	H	-0.6292770	-0.2331240	2.4233560
58	No	Show	58	H	-1.8890550	0.0150530	-1.5294640
59	No	Show	59	H	-0.4033300	0.8268110	-1.0793720
60	No	Show	60	H	-2.3988180	-1.2341080	1.8977040
61	No	Show	61	H	-3.4674130	-0.0572520	1.1662980
62	No	Show	62	H	-0.3121900	3.1610720	-0.8538230
63	No	Show	63	H	-1.4631220	5.3709750	-0.9818290
64	No	Show	64	H	-3.9896020	5.4387070	-0.9665310
65	No	Show	65	H	-5.2341490	3.2817160	-0.8567300
66	No	Show	66	H	-5.5673570	-2.0742840	-2.2687360
67	No	Show	67	H	-4.7342870	-4.4330620	-2.3730710
68	No	Show	68	H	-2.7734350	-5.0857180	-0.9273090
69	No	Show	69	H	-1.7462700	-3.3748550	0.5840630
70	No	Show	70	Zn	-4.4820670	0.4186350	-0.7236180

Optimized (B3LYP/cc-pVDZ) ground-state geometries of PQPc+Zn^{2+} in ACN.

Row	Highlight	Display	Tag	Symbol	X	Y	Z
1	No	Show	1	C	1.1961960	4.4727480	2.3217990
2	No	Show	2	C	2.4797950	4.7752590	1.7928040
3	No	Show	3	C	3.1930200	3.8076340	1.1270540
4	No	Show	4	C	2.6692940	2.4905550	0.9613520
5	No	Show	5	C	1.3547890	2.1742610	1.4808130
6	No	Show	6	C	0.6510510	3.2194880	2.1628270
7	No	Show	7	N	3.4404060	1.5982890	0.2883980
8	No	Show	8	C	2.9596750	0.3732370	0.2303740
9	No	Show	9	C	1.7089870	-0.0962610	0.7633330
10	No	Show	10	C	0.8307980	0.8526990	1.3009050
11	No	Show	11	N	3.5744440	-0.7174200	-0.3623750
12	No	Show	12	N	2.8356980	-1.8434590	-0.1604590
13	No	Show	13	C	1.7365450	-1.5250690	0.5016980
14	No	Show	14	C	0.8840160	-2.6430500	0.9642290
15	No	Show	15	C	4.8357260	-0.8224220	-1.0152670
16	No	Show	16	C	0.4302140	-2.7048090	2.2939700
17	No	Show	17	C	-0.2393120	-3.8384210	2.7652780
18	No	Show	18	C	-0.4575090	-4.9312680	1.9188090
19	No	Show	19	C	-0.0080400	-4.8789640	0.5936660
20	No	Show	20	C	0.6558850	-3.7454310	0.1199370
21	No	Show	21	C	5.5114670	0.3192440	-1.4762200
22	No	Show	22	C	6.7425790	0.1752090	-2.1225600
23	No	Show	23	C	7.3059040	-1.0881040	-2.3227160
24	No	Show	24	C	6.6231570	-2.2201250	-1.8641890

25	No	Show	25	C	5.3962850	-2.0960900	-1.2107670
26	No	Show	26	C	-0.6106070	0.5333690	1.6491060
27	No	Show	27	N	-1.3712960	-0.1253970	0.5655020
28	No	Show	28	C	-1.3649230	0.5393930	-0.7491840
29	No	Show	29	C	-2.6989040	-0.5036310	1.0604860
30	No	Show	30	C	-1.8642330	1.9812830	-0.8610870
31	No	Show	31	C	-3.3985020	-1.5407130	0.2049480
32	No	Show	32	C	-0.9417390	3.0275390	-0.9467140
33	No	Show	33	C	-1.3694640	4.3502500	-1.0524540
34	No	Show	34	C	-2.7375070	4.6158390	-1.0683520
35	No	Show	35	C	-3.6173590	3.5450160	-1.0028290
36	No	Show	36	N	-3.2001180	2.2573980	-0.9110280
37	No	Show	37	N	-4.3662150	-1.1378420	-0.6577550
38	No	Show	38	C	-5.0400670	-2.0455210	-1.4029610
39	No	Show	39	C	-4.7721100	-3.4046320	-1.3198650
40	No	Show	40	C	-3.7676830	-3.8344830	-0.4499150
41	No	Show	41	C	-3.0791800	-2.8939800	0.3152640
42	No	Show	42	H	0.6385760	5.2400400	2.8620290
43	No	Show	43	H	2.8978510	5.7757600	1.9210010
44	No	Show	44	H	4.1831790	4.0076370	0.7144810
45	No	Show	45	H	-0.3289990	3.0228100	2.5942880
46	No	Show	46	H	0.6465480	-1.8849810	2.9812320
47	No	Show	47	H	-0.5732550	-3.8743540	3.8044510
48	No	Show	48	H	-0.9719380	-5.8200090	2.2904020
49	No	Show	49	H	-0.1732660	-5.7272020	-0.0742690
50	No	Show	50	H	1.0152010	-3.7096710	-0.9095780
51	No	Show	51	H	5.0798720	1.3039690	-1.3176150
52	No	Show	52	H	7.2614840	1.0690270	-2.4760410
53	No	Show	53	H	8.2671320	-1.1905860	-2.8302510
54	No	Show	54	H	7.0494330	-3.2150630	-2.0103930
55	No	Show	55	H	4.8637500	-2.9743770	-0.8509610
56	No	Show	56	H	-1.1247690	1.4464870	1.9897640
57	No	Show	57	H	-0.6314640	-0.1613600	2.5026250
58	No	Show	58	H	-1.9324420	-0.0981290	-1.4423660
59	No	Show	59	H	-0.3311360	0.5325800	-1.1189740
60	No	Show	60	H	-2.5586100	-0.9464220	2.0569840
61	No	Show	61	H	-3.3658750	0.3694150	1.2255350
62	No	Show	62	H	0.1217440	2.7951790	-0.9386360
63	No	Show	63	H	-0.6414150	5.1596920	-1.1236680
64	No	Show	64	H	-3.1281070	5.6301130	-1.1452590
65	No	Show	65	H	-4.6952400	3.7087560	-1.0354450
66	No	Show	66	H	-5.8032910	-1.6514370	-2.0745660
67	No	Show	67	H	-5.3401700	-4.1056450	-1.9308500
68	No	Show	68	H	-3.5254800	-4.8948420	-0.3623260
69	No	Show	69	H	-2.2969460	-3.2012730	1.0073620
70	No	Show	70	Zn	-4.6060760	0.8177540	-0.9875460

Optimized (B3LYP/cc-pVDZ) excited-state geometries of PQPc+Zn^{2+} in ACN.

Row	Highlight	Display	Tag	Symbol	X	Y	Z

1	No	Show	1	C	1.3285900	4.4307290	2.5618540
2	No	Show	2	C	2.5753130	4.7248610	1.9989920
3	No	Show	3	C	3.2413780	3.7414410	1.2612340
4	No	Show	4	C	2.6873400	2.4603460	1.0694170
5	No	Show	5	C	1.3860590	2.1504260	1.6234030
6	No	Show	6	C	0.7502510	3.1710970	2.3776210
7	No	Show	7	N	3.4246040	1.5486840	0.3301240
8	No	Show	8	C	2.9004890	0.3427510	0.2799620
9	No	Show	9	C	1.6842200	-0.1241280	0.8291800
10	No	Show	10	C	0.7998140	0.8470300	1.3898030
11	No	Show	11	N	3.4943570	-0.7807900	-0.3702040
12	No	Show	12	N	2.7620460	-1.9113100	-0.1751830
13	No	Show	13	C	1.7038430	-1.5472240	0.5808010
14	No	Show	14	C	0.8976850	-2.6345040	1.1294420
15	No	Show	15	C	4.6609480	-0.8575750	-1.1168540
16	No	Show	16	C	0.3200990	-2.5401130	2.4144960
17	No	Show	17	C	-0.3345960	-3.6343960	2.9788330
18	No	Show	18	C	-0.4341050	-4.8398930	2.2705820
19	No	Show	19	C	0.1358540	-4.9468990	0.9937820
20	No	Show	20	C	0.8045670	-3.8623720	0.4322480
21	No	Show	21	C	5.4842770	0.2816110	-1.3237080
22	No	Show	22	C	6.6462410	0.1528190	-2.0725820
23	No	Show	23	C	7.0112770	-1.0840120	-2.6255790
24	No	Show	24	C	6.1948800	-2.2108490	-2.4229970
25	No	Show	25	C	5.0306010	-2.1111010	-1.6794810
26	No	Show	26	C	-0.6651210	0.6051100	1.6395030
27	No	Show	27	N	-1.3714850	-0.0796990	0.5245310
28	No	Show	28	C	-1.2916170	0.5841980	-0.7881460
29	No	Show	29	C	-2.7281640	-0.4520420	0.9301780
30	No	Show	30	C	-1.8480060	2.0027360	-0.9056460
31	No	Show	31	C	-3.3211590	-1.5337060	0.0543450
32	No	Show	32	C	-0.9661870	3.0856880	-0.8248710
33	No	Show	33	C	-1.4308410	4.3956990	-0.9189270
34	No	Show	34	C	-2.7968570	4.6118450	-1.0950800
35	No	Show	35	C	-3.6332400	3.5083680	-1.1789950
36	No	Show	36	N	-3.1822030	2.2308470	-1.0912770
37	No	Show	37	N	-4.2168540	-1.1930910	-0.9073270
38	No	Show	38	C	-4.7689570	-2.1513780	-1.6918080
39	No	Show	39	C	-4.4490300	-3.4930350	-1.5518690
40	No	Show	40	C	-3.5185510	-3.8585820	-0.5750670
41	No	Show	41	C	-2.9534020	-2.8702630	0.2270590
42	No	Show	42	H	0.8002930	5.1812250	3.1540790
43	No	Show	43	H	3.0296010	5.7079810	2.1374610
44	No	Show	44	H	4.2170240	3.9409360	0.8131740
45	No	Show	45	H	-0.2105030	2.9697430	2.8521200
46	No	Show	46	H	0.4382790	-1.6243470	2.9927140
47	No	Show	47	H	-0.7583140	-3.5527060	3.9816150
48	No	Show	48	H	-0.9483040	-5.6947130	2.7145150
49	No	Show	49	H	0.0621160	-5.8845520	0.4393050
50	No	Show	50	H	1.2580050	-3.9449940	-0.5556030
51	No	Show	51	H	5.1836900	1.2308270	-0.8886470

52	No	Show	52	H	7.2783490	1.0279820	-2.2307790
53	No	Show	53	H	7.9272050	-1.1720710	-3.2121800
54	No	Show	54	H	6.4783950	-3.1736180	-2.8509160
55	No	Show	55	H	4.3927230	-2.9740570	-1.5071520
56	No	Show	56	H	-1.1558640	1.5655060	1.8791570
57	No	Show	57	H	-0.8349850	-0.0377050	2.5201500
58	No	Show	58	H	-1.7627460	-0.0711320	-1.5367890
59	No	Show	59	H	-0.2297150	0.6371390	-1.0616800
60	No	Show	60	H	-2.6643930	-0.8522910	1.9525580
61	No	Show	61	H	-3.4160060	0.4177310	1.0012640
62	No	Show	62	H	0.0956440	2.8869640	-0.6884400
63	No	Show	63	H	-0.7344000	5.2334650	-0.8577750
64	No	Show	64	H	-3.2178310	5.6137510	-1.1748930
65	No	Show	65	H	-4.7060310	3.6381070	-1.3272850
66	No	Show	66	H	-5.4803930	-1.8141520	-2.4460050
67	No	Show	67	H	-4.9204360	-4.2305790	-2.2009190
68	No	Show	68	H	-3.2394030	-4.9044030	-0.4373370
69	No	Show	69	H	-2.2280010	-3.1233710	0.9982790
70	No	Show	70	Zn	-4.5609070	0.7394980	-1.2945170
